# Visualizing the non-equilibrium dynamics of photoinduced intramolecular electron transfer with femtosecond X-ray pulses

**DOI:** 10.1038/ncomms7359

**Published:** 2015-03-02

**Authors:** Sophie E. Canton, Kasper S. Kjær, György Vankó, Tim B. van Driel, Shin-ichi Adachi, Amélie Bordage, Christian Bressler, Pavel Chabera, Morten Christensen, Asmus O. Dohn, Andreas Galler, Wojciech Gawelda, David Gosztola, Kristoffer Haldrup, Tobias Harlang, Yizhu Liu, Klaus B. Møller, Zoltán Németh, Shunsuke Nozawa, Mátyás Pápai, Tokushi Sato, Takahiro Sato, Karina Suarez-Alcantara, Tadashi Togashi, Kensuke Tono, Jens Uhlig, Dimali A. Vithanage, Kenneth Wärnmark, Makina Yabashi, Jianxin Zhang, Villy Sundström, Martin M. Nielsen

**Affiliations:** 1 Department of Synchrotron Radiation Instrumentation, Lund University, PO Box 118, 22100 Lund, Sweden; 2 Centre for Molecular Movies, Niels Bohr Institute, University of Copenhagen, DK-2100 Copenhagen, Denmark; 3 Centre for Molecular Movies, Department of Physics, Technical University of Denmark, DK-2800 Lyngby, Denmark; 4 Wigner Research Centre for Physics, Hungarian Academy Sciences, PO Box 49, H-1525 Budapest, Hungary; 5 High Energy Accelerator Research Organization, 1-1 Oho, Tsukuba, Ibaraki 305-0801, Japan; 6 European XFEL, Albert-Einstein Ring 19, D-22761, Hamburg, Germany; 7 The Hamburg Centre for Ultrafast Imaging, Luruper Chaussee 149, 22761 Hamburg, Germany; 8 Department of Chemical Physics, Lund University, PO Box 124, S-22100 Lund, Sweden; 9 Department of Chemistry, Technical University of Denmark, DK-2800, Kongens Lyngby, Denmark; 10 Center for Nanoscale Materials, Argonne National Laboratory, 9700 South Cass Avenue, Argonne, Illinois 60439, USA; 11 Department of Chemistry, Centre for Analysis and Synthesis, Lund University, S-22100 Lund, Sweden; 12 RIKEN SPring-8 Center, 1-1-1 Kouto, Sayo-cho, Sayo-gun, Hyogo 679-5148, Japan; 13 Japan Synchrotron Radiation Research Institute (JASRI), 1-1-1 Kouto, Sayo-cho, Sayo-gun, Hyogo 679-5198, Japan

## Abstract

Ultrafast photoinduced electron transfer preceding energy equilibration still poses many experimental and conceptual challenges to the optimization of photoconversion since an atomic-scale description has so far been beyond reach. Here we combine femtosecond transient optical absorption spectroscopy with ultrafast X-ray emission spectroscopy and diffuse X-ray scattering at the SACLA facility to track the non-equilibrated electronic and structural dynamics within a bimetallic donor–acceptor complex that contains an optically dark centre. Exploiting the 100-fold increase in temporal resolution as compared with storage ring facilities, these measurements constitute the first X-ray-based visualization of a non-equilibrated intramolecular electron transfer process over large interatomic distances. Experimental and theoretical results establish that mediation through electronically excited molecular states is a key mechanistic feature. The present study demonstrates the extensive potential of femtosecond X-ray techniques as diagnostics of non-adiabatic electron transfer processes in synthetic and biological systems, and some directions for future studies, are outlined.

## Abstract (web summary)

Photoinduced electron transfer in solvated molecular assemblies occurs on the ultrafast timescale before full electronic and geometric relaxation take place. Here Canton *et al.* monitor this out-of-equilibrium process in a donor–acceptor bimetallic assembly using an X-ray free-electron laser.

## Graphical abstract

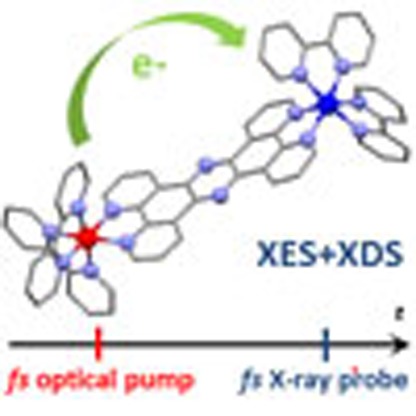

Photoinduced electron transfer (ET) is one of the primary events associated with the functionality of solvated molecular assemblies, ranging from simple dyads to large proteins. Studies across physics, chemistry and biology have been rationalizing how structure and surroundings are shaping the free-energy landscape for this fundamental process^1^. The respective influence of intra- and intermolecular factors can often be readily disentangled for ET involving equilibrated reactants^2^. This is no longer the case on the ultrafast timescale, since neither the nuclear degrees of freedom nor the first solvation shell has yet adapted to the electronic configuration of the nascent excited state^3^. The photoinduced charge migration is thus proceeding out of equilibrium. The fundamental importance and practical relevance of this regime have both emerged from the novel results obtained with multidimensional optical spectroscopies, which have been enabled by the progress in femtosecond laser technologies^4^,^5^,^6^,^7^. The relaxation from the Franck–Condon state to the lowest thermally equilibrated excited state can be generally described in terms of interligand ET, intramolecular vibrational relaxation, internal conversion and intersystem crossing^8^,^9^. Investigating the interplay between these deactivation pathways in simple mononuclear complexes has been geared intensively towards optimizing the excited-state properties of light emitters and sensitizers, for example, the versatile families of polypyridyl compounds derived from [Ir^II^(ppy)_3_], [Ru^II^(bpy)_3_] or [Os^II^(bpy)_3_]^10^,^11^,^12^. As light-harvesting building blocks, they can be integrated to photomolecular devices that transform solar energy into electrical or chemical potential via ultrafast ET. For such functional complexes, maximizing the yields of long-lived charge-separated species, while minimizing adverse heat dissipation are highly complementary goals pursued to increase their selectivity and stability, hence their overall efficiency. Ultimately, controlling the dynamics of non-equilibrated ET would enable harnessing the largely untapped potential of ‘hot’ transitions for driving numerous photoconversion schemes with significant excess of stored electronic energy^13^,^14^,^15^.

Deciphering the dynamics of intramolecular ET as the system relaxes and thermalizes requires mapping the spatial and temporal redistribution of the energy deposited initially by photoabsorption. So far, this problem has been tackled with the spectroscopic tools developed for monitoring solvation dynamics and vibronic cooling in large molecules^16^. This usually relies on correlating information gathered from measurements conducted separately in different spectral regions with varying experimental conditions (for example, solute concentrations or laser fluences). A salient complication faced in the ultraviolet–visible and near infrared range is the low degree of element- and spin specificity displayed by optical transitions, which have to obey strict dipole selection rules. The applicability of these spectroscopies is thus restricted to participating states that are optically bright. In addition, the dynamics of non-equilibrated ET often exhibit distinctive excitation wavelength dependencies and multi- or non-exponential kinetic behaviours. Although the interpretation can be assisted by density functional theory (DFT), time-dependent DFT and molecular dynamics (MD) simulations^3^, the current understanding of the process needs to be refined down to the molecular level before descriptive and predictive models can be firmly validated. The exploration of this conceptual frontier is anticipated to advance rapidly at X-ray free-electron laser (XFEL) facilities, where optical pump-X-ray probe detection schemes can now track the electronic and structural dynamics on the atomic scale with femtosecond resolution in the gas^17^, solution^18^,^19^ and solid phase^20^,^21^,^22^.

Here we combine X-ray Emission Spectroscopy (XES) and X-ray Diffuse Scattering (XDS) with the highly intense ultrashort pulses delivered by the SACLA XFEL facility^23^ to address the long-standing challenge of characterizing non-equilibrated ET in donor–acceptor assemblies that contain optically dark active sites. The bimetallic complex studied in this work consists of a light-harvesting, ruthenium (Ru)-based chromophore linked to an optically dark cobalt (Co) electron sink by a bridge that mediates ultrafast ET. This prototypical dyad exemplifies the wide class of synthetic and natural photocatalysts for which the coupled electronic and structural dynamics are only partially understood beyond the decay of the Franck–Condon state. In the present study, Co Kα_1_ XES based on 2*p*–1*s* electronic transitions is employed to follow the ET from the photoexcited Ru centre to the Co centre with inherent element- and spin sensitivity^24^,^25^,^26^. Analysis of the azimuthally integrated one-dimensional X-ray scattering signals *S*(*Q*), as a function of the momentum transfer *Q* is used to extract the phototriggered structural changes and the kinetics of thermalization^27^,^28^,^29^,^30^,^31^,^32^. Going beyond the limited information delivered by transient optical absorption spectroscopy, these simultaneous XFEL experiments retrieve the timescales that are necessary to fully describe the non-equilibrated ET in the photoexcited dyad. The immediate prospects offered by this general methodology to the diagnostics and optimization of ‘hot’ photoactive molecular complexes are further highlighted.

## Results

### Tracking the optically bright dynamics in photoexcited RuCo

**Figure 1:**
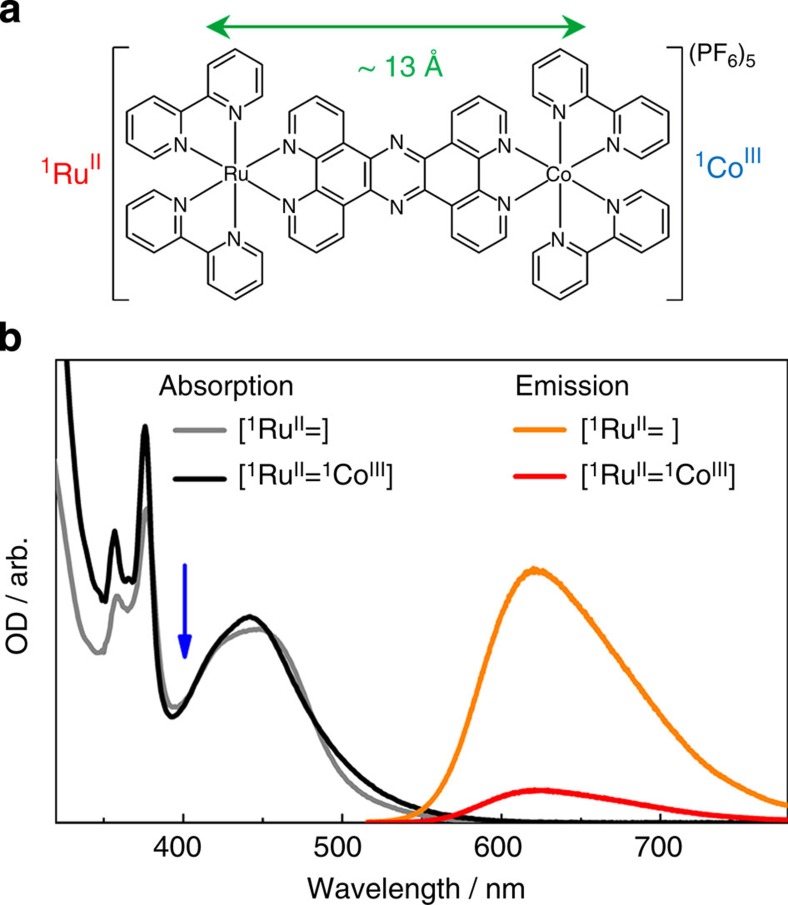
The [^1^Ru^II^=^1^Co^III^] complex. (**a**) The molecular structure of the dyad studied in this work. The Ru and Co centres are held 13 Å apart by the tpphz rigid bridge. (**b**) Absorption and emission spectra of [^1^Ru^II^=] and [^1^Ru^II^=^1^Co^III^] in acetonitrile. The pump wavelength used for all the optical and X-ray experiments is indicated by the blue arrow.

**Figure 2:**
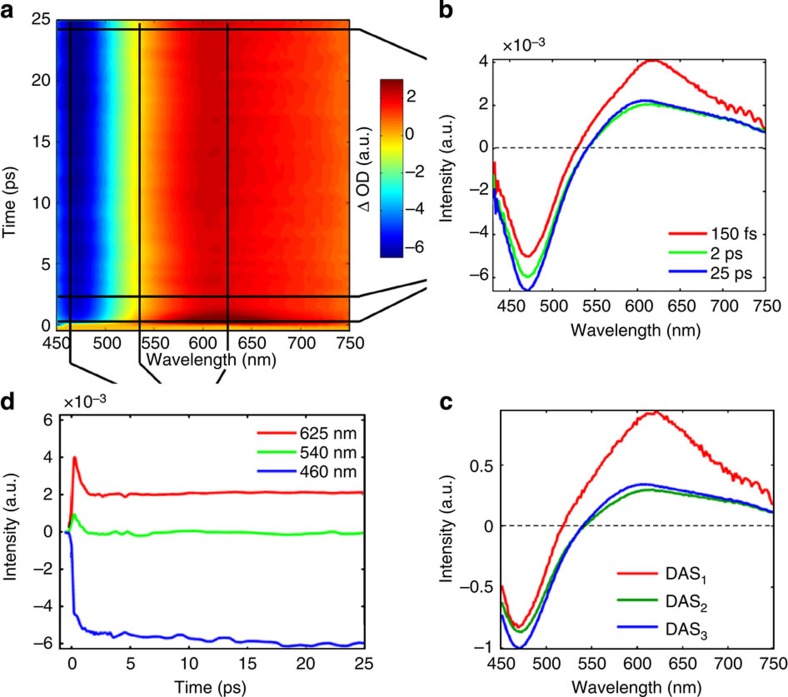
Ultrafast optical absorption spectroscopy. Clockwise: (**a**) Transient optical absorption spectra of [^1^Ru^II^=^1^Co^III^] excited at 400 nm as a function of pump-probe time delay. (**b**) Transient absorption spectra at three pump-probe time delays: 150 fs, and 2 and 25 ps. (**c**) The three decay-associated spectra DAS_1_, DAS_2_ and DAS_3_ returned by the global analysis fitting procedure (GA-fit). (**d**) Kinetic traces over the first 25 ps at three different probe wavelengths: 460, 540 and 625 nm.

The bimetallic RuCo complex [(bpy)_2_^1^Ru^II^(tpphz)^1^Co^III^(bpy)_2_]^5+^ (with bpy=bipyridine, tpphz=tetrapyrido (3,2-*a*:2′ 3′ -*c*:3′′,2′′ -*h*::2′′′,3′′′-*j*) phenazine)^33^ studied in this work is shown in Fig. 1a. Its PF_6_ salt was synthesized following an improved protocol^34^. This dyad consists of a ^1^Ru^II^ (t_2g_)^6^ centre and a ^1^Co^III^ (t_2g_)^6^ centre in the low-spin (LS) state, which are held apart at fixed distance (~13 Å) and orientation by the rigid bridge tpphz. This planar π-conjugated system is known to act as a ‘molecular wire’ capable of mediating ultrafast ET^35^. The chemical structure is abbreviated as [^1^Ru^II^=^1^Co^III^ (LS)] hereafter. The steady-state optical absorption spectrum of the [(bpy)_2_^1^Ru^II^(tpphz)]^2+^ moiety (denoted [^1^Ru^II^=]) in acetonitrile (MeCN) is displayed in Fig. 1b. The tpphz ligand-centred transitions and the two singlet metal-to-ligand charge transfer (MLCT) transitions Ru→bpy and Ru→tpphz give rise, respectively, to the absorbance in the 340–380, 400–440 and 440–550 nm regions^36^,^37^. The steady-state emission spectrum of [^1^Ru^II^=] excited at 400 nm (Fig. 1b) closely resembles that from the ^3^MLCT in [Ru^II^(phen)_3_]^35^,^36^,^37^ (where phen=phenanthroline) with a maximum intensity at 622 nm. Although the optical absorption spectrum of [^1^Ru^II^=^1^Co^III^ (LS)] does not change appreciably after coordination of the ^1^Co^III^ moiety, its emission yield is reduced by >80% without noticeable alteration in the spectral lineshape. The dynamics of this quenching process can be followed with femtosecond transient optical absorption spectroscopy (TOAS) as shown in Fig. 2a (Supplementary Note 1). On selective photoexcitation of the ^1^MLCT in the Ru chromophore at 400 nm, a band peaking at around 625 nm appears quasi-instantaneously and decays very rapidly (red trace in Fig. 2b). After 25 ps, the transient absorption spectrum has evolved into a broader band of weaker intensity (blue trace in Fig. 2b). A global-fit analysis of the transient absorption signal within the first 30 ps reveals that three Decay-Associated Spectra DAS_1_, DAS_2_ and DAS_3_ (Fig. 2c) are needed to describe adequately the dynamics. The details of this analysis are outlined in the Supplementary Note 1. The reduced pyrazine state^35^,^36^,^37^ (DAS_1_) decays with a 490±17 fs lifetime to a hot ^3^MLCT (DAS_2_) reminiscent of the ^3^MLCT in [Ru(phen)_3_]^2+^ (refs 35, 36, 37). The cooling to the thermalized excited state occurs with an 8±3 ps time constant (DAS_3_). Figure 2d shows the kinetics traces at 460, 540 and 625 nm. A model accounting for the steady-state and time-resolved spectroscopic observations can be built, where the relaxation of the Franck–Condon state branches out into two parallel intramolecular ET pathways (Supplementary Fig. 1). In the first route, the bridge-localized CT state [(bpy)_2_^2^Ru^III^(tpphz)·^1^Co^III^(bpy)_2_]^5+^ (denoted [^2^Ru^III^(=·)^1^Co^III^ (LS)]) is populated instantaneously (<50 fs) and decays on the sub-picosecond timescale. In the second route, the phen-like portion of the tpphz ligand is formally reduced. This is the state that gives rise to the quenched steady-state emission observed in Fig. 1b. It should be noted that such a model holds when the pump wavelength is varied across the absorption band between 370 and 480 nm (Supplementary Fig. 2). All the extracted time constants exhibit the pronounced excitation wavelength dependency that could be expected from an intermolecular ET event proceeding out of equilibrium, as mentioned in the introduction. From these TOAS measurements, it is clearly possible to determine the residence time of the electron on the bridge. However, the dynamics that take place at the Co centre completely elude detection. The deceptively simple questions ‘when is the electron reaching the ^1^Co^III^ centre?’, and ‘how does the relaxation at the reduced Co^II^ centre take place?’ remain unanswered. Very similar unresolved issues about the actual electronic localization and phototriggered structural dynamics are encountered in the studies of fully functional photoactive materials (for example, homogeneous or heterogeneous photocatalysts^38^,^39^,^40^,^41^ and organic–inorganic hybrid solar cells^42^,^43^) whenever weak optically bright or optically dark transitions play a role in the photoconversion process. This particular [^1^Ru^II^=^1^Co^III^ (LS)] compound is therefore a very well-suited candidate to demonstrate the unique capabilities of ultrafast X-ray techniques for investigating such processes. As shown below, femtosecond XES and XDS are successfully employed in this work to capture the optically dark electronic and structural dynamics that take place within the photoexcited donor–bridge–acceptor complex.

### Tracking the optically dark dynamics in photoexcited RuCo

**Figure 3:**
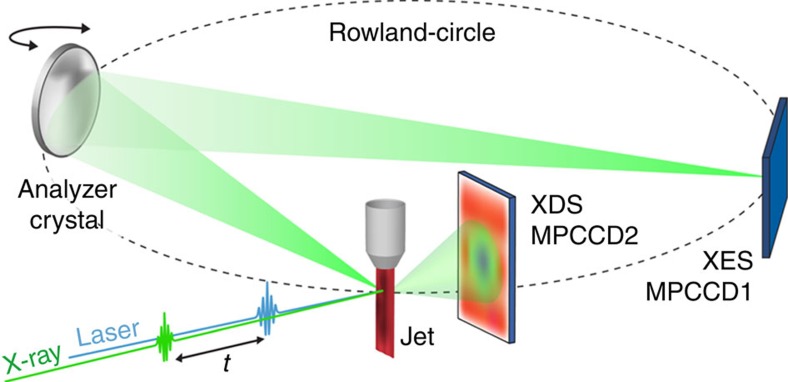
Experimental setup. This optical pump-X-ray probe detection scheme combining XES and XDS on photoexcited species in solution was implemented at the SACLA XFEL facility.

**Figure 4:**
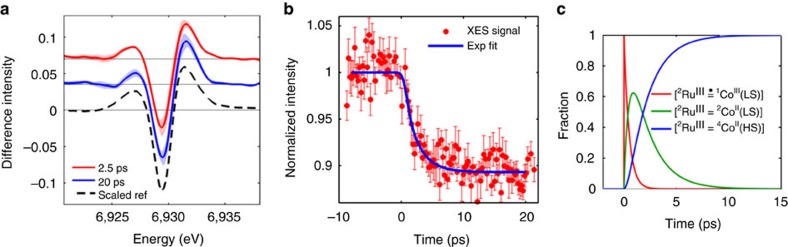
Ultrafast X-ray emission spectroscopy. (**a**) Co Kα_1_ Δ*S*_XES_(*t*) at 2.5 (red) and 20 ps (blue) pump-probe delay. The shaded areas indicate the uncertainty level. The dashed black curve is the simulated reference for a ^1^Co^III^(LS)→^4^Co^II^(HS) conversion, scaled to the 20 ps trace. (**b**) Kinetic trace at 6.93 keV (red dots) and single-exponential fit with a 1.9 ps lifetime, broadened by a 520±410 fs XFEL IRF (blue line). The error bars indicate the s.e. of each data point. (**c**) Time evolution for the fractions of [^2^Ru^III^(=·)^1^Co^III^ (LS)] (red), [^2^Ru^III^=^2^Co^II^(LS)] (green) and [^2^Ru^III^=^4^Co^II^(HS)] (blue) as monitored by the combination of femtosecond TOAS and XES, where the initial fraction of [^2^Ru^III^(=·)^1^Co^III^ (LS)] was renormalized to 1.

A pump-probe setup accommodating simultaneous XES and XDS measurements^44^,^45^ was implemented at BL3 of the SACLA XFEL (Fig. 3). Figure 4a displays the Co Kα_1_ XES difference signal Δ*S*_XES_(*t*) [laser_ON_ (*t*)−laser_OFF_] acquired at time delays *t* fixed to 2.5 (red trace) and 20 ps (blue trace) after the selective excitation at 400 nm of the ^1^MLCT state in the Ru^II^ moiety (Supplementary Note 2; Supplementary Fig. 3). This X-ray emission line originates from the secondary 2*p*_3/2_→1*s* transition subsequent to 1*s* core ionization. Steady-state^24^,^26^,^46^ and time-resolved^45^,^47^,^48^ experiments at storage ring facilities have established that for spin-state transitions (SSTs) in 3*d* transition metal ions, the full width at half maximum (FWHM) of the Kα_1_ lines (hence its inverse maximum intensity) is directly proportional to the number of unpaired electrons. This spectral feature therefore carries information about the total spin momentum of the X-ray absorbing centre. To assign the transient signal Δ*S*_XES_(*t*), the spectra were compared with a static reference trace constructed by subtracting the normalized lineshapes measured for a (πt_2g_)^6^ [^1^Co^III^] centre in the LS state from that of a (πt_2g_)^5^ (σe_g_)^2^ [^4^Co^II^] centre in the high-spin (HS) state (Supplementary Note 3; Supplementary Fig. 4). The strong similarity with the Δ*S*_XES_ at *t*=20 ps demonstrates that ET from Ru^II^* to ^1^Co^III^ (LS) and a SST at the reduced Co^II^ centre have both taken place during this time interval. Such finding concurs with previous synchrotron-based transient X-ray absorption experiments, where the reduced Co^II^ moiety was observed in the quartet HS state^34^ with an 80 ps temporal resolution. Relative scaling of the reference trace (black dashed trace in Fig. 4a) to Δ*S*_XES_ at *t*=20 ps delivers 65±8% as the fraction of [^2^Ru^III^=^4^Co^II^(HS)]. It depends both on the initial concentration of photoexcited [^1^Ru^II^*=^1^Co^III^] and on the yield of [^2^Ru^III^=^4^Co^II^ (HS)] population via ET and SST. By the above-mentioned relation between X-ray emission intensity and spin state, the amplitude of Δ*S*_XES_(*t*) at 6.93 keV, *γ*_XES_(*t*), monitors the formation kinetics of the charge-separated state where the reduced Co^II^ species is in the HS state. Within the signal to noise of this experiment, the appearance of this species clearly occurs on the few picosecond timescale (red dots in Fig. 4b).

The electronic dynamics in the photoexcited dyad as followed by XES can now be contrasted to that observed with TOAS. While the TOAS signal has almost completely decayed at 2 ps, the XES difference signal is approaching its maximum amplitude only at 10 ps. In other words, the time taken by the electron to leave the bridge (490±17 fs) cannot be identified with the time necessary for the [^2^Ru^III^=^4^Co^II^ (HS)] to appear (few picosecond). On leaving the bridge, the electron could localize on the distal portion of tpphz as a reduced ligand state or on the Co centre as a metal-centred state. Since no dynamics associated with the spectral fingerprints of bpy^−^ or phen^−^ (ref. 49) can be observed with TOAS in the ultraviolet and visible range, the intermediate species is optically dark. A sequential reaction mechanism involving the ^2^Co^II^ (LS) electronically excited state can then be proposed, namely [^1^Ru^II^=^1^Co^III^ (LS)]+*hν*→[^2^Ru^III^(=·)^1^Co^III^ (LS)]→[^2^Ru^III^=^2^Co^II^(LS)]→[^2^Ru^III^=^4^Co^II^ (HS)]. Within such a model, the formation rate of the intermediate species is locked to the (490 fs)^−1^ decay rate of reduced pyrazine. The ratio of the XES amplitudes for [^2^Ru^III^=^2^Co^II^(LS)]/[^2^Ru^III^=^4^Co^II^ (HS)] is fixed to 1/3, which would be expected from the variations in FWHM for the XES signal of a ^2^Co^II^ (LS)→^4^Co^II^(HS) SST. Fitting the kinetics trace *γ*_XES_(*t*) (blue line in Fig. 4b, Table 1 in the Methods section and Supplementary Note 3) with the width of the Gaussian XFEL instrument response function (IRF) as a free parameter returns 1.9±0.6 ps for the time constant of the step [^2^Ru^III^=^2^Co^II^(LS)]→[^2^Ru^III^=^4^Co^II^ (HS)], and 520±410 fs for the IRF, which is dominated by the temporal jitter between optical pump and X-ray probe. Since the measured ET rate is not instrument limited^50^, the XES experiment is accessing the intrinsic timescale of the process. The temporal evolution of the [^2^Ru^III^(=·)^1^Co^III^ (LS)], [^2^Ru^III^=^2^Co^II^ (LS)] and [^2^Ru^III^=^4^Co^II^ (HS)] populations are shown in Fig. 4c (Supplementary Note 5). Summarizing the first conclusion reached in this study: the combination of femtosecond XES and TOAS measurements demonstrates that sub-picosecond ET occurs from the photoexcited Ru centre to an electronically excited state of the Co^II^ centre, tentatively assigned as ^2^Co^II^(LS). This is followed by an ~2 ps electron localization, resulting in the formation of the [^2^Ru^III^=^4^Co^II^ (HS)] charge-separated species. Since these timescales are typical of intramolecular vibration, energy redistribution and heat dissipation from vibrationally hot states to the environment, the complete characterization of the charge localization requires obtaining further information about the global structural changes and the interaction of the complex with its surroundings. Whereas this usually challenges optical spectroscopies, both aspects are readily amenable to investigation by the analysis of the XDS difference signal Δ*S*_XDS_(*Q,t*) acquired simultaneously with Δ*S*_XES_(*t*) in the present experiments.

**Figure 5:**
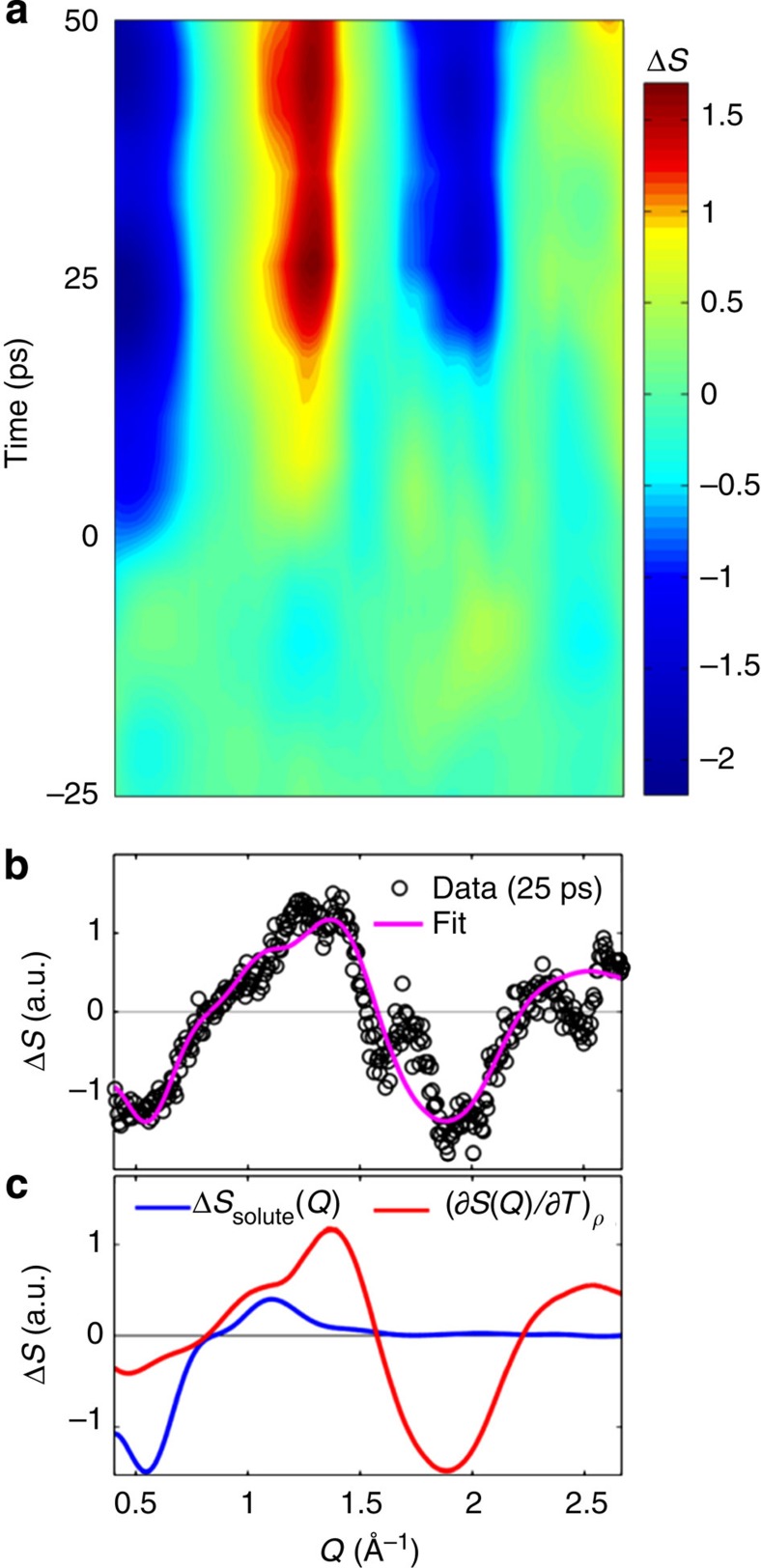
Ultrafast X-ray diffuse scattering. (**a**) Median filtered Δ*S*_XDS_(*Q,t*). (**b**) Experimental (black dots) and fitted (purple line) Δ*S*_XDS_(*Q,*25 ps). (**c**) Contributions from the solute (blue) and from the solvent (red).

**Figure 6:**
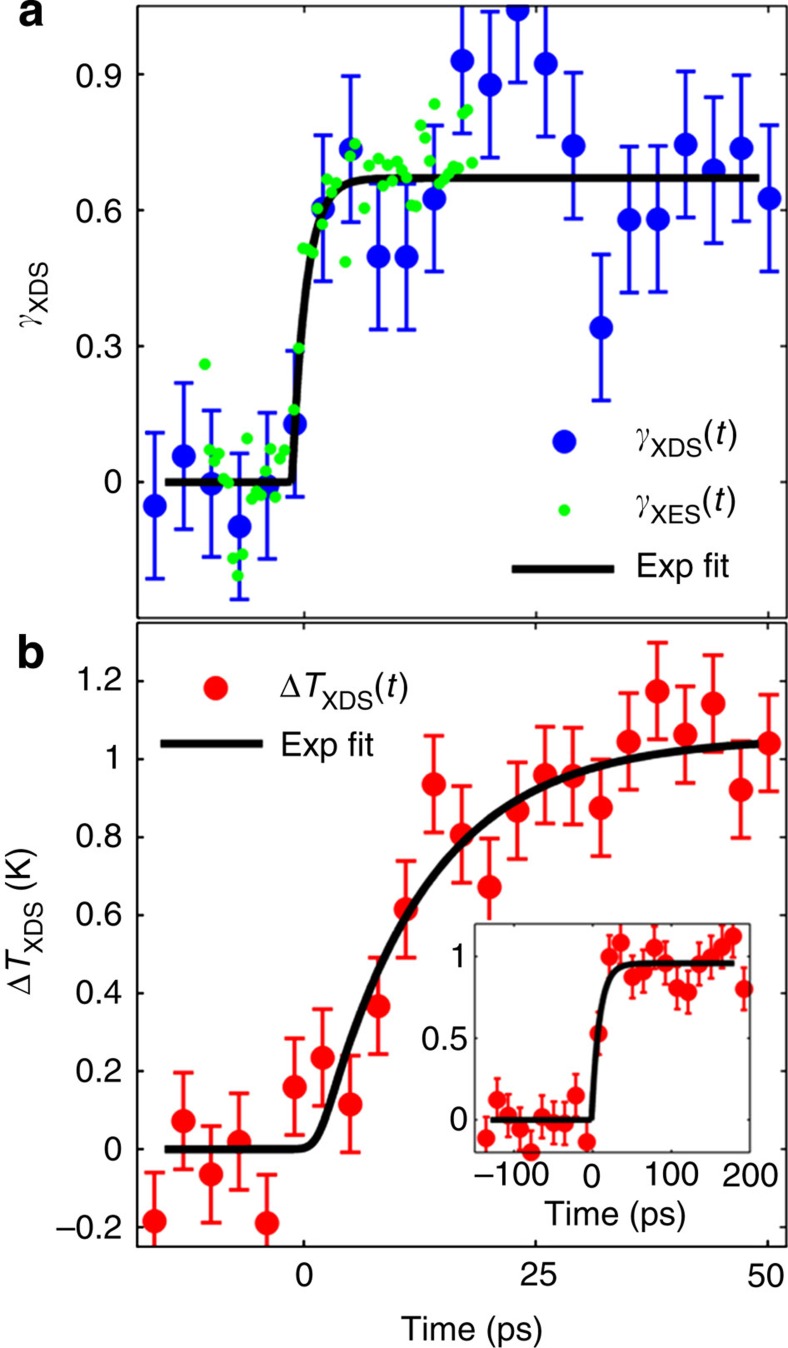
Ultrafast XDS kinetics. (**a**) *γ*_XDS_(*t*) (blue dots) and *γ*_XES_(*t*) (green dots) as a function of pump-probe time delay. The single-exponential fits of *γ*_XDS_(*t*) is indicated by the black line. (**b**) Δ*T*(*t*) kinetics (red dots), with its single-exponential fit (black line). The error bars six indicate the s.d. of the data points.

Figure 5a shows the measured Δ*S*_XDS_(*Q,t*) after data reduction and background subtraction^51^ (Supplementary Notes 5 and 6; Supplementary Fig. 6). This signal is interpreted as arising primarily from the changes in solute structure Δ*S*_solute_(*Q,t*) and from the bulk-solvent response Δ*S*_solvent_(*Q,t*) (Supplementary Note 7). The contribution Δ*S*_solute_(*Q,t*) can be expressed as *γ*_XDS_(*t*) × Δ*S*_DFT_(*Q*), where *γ*_XDS_(*t*) is the time-dependent fraction of [^2^Ru^III^=^4^Co^II^ (HS)], and Δ*S*_DFT_(*Q*) is the profile calculated from the spin unrestricted DFT-optimized geometries of solvated [^1^Ru^II^=^1^Co^III^ (LS)] and [^2^Ru^III^=^4^Co^II^ (HS)] (Supplementary Note 4). The distinctive negative dip at *Q*=0.5 Å^−1^ is associated to the Co–N bond length elongation by Δ*R*~0.2 Å in the ^4^Co^II^ HS state (Supplementary Fig. 7; Supplementary Fig. 10)^34^. For the time delays *t*>3 ps considered here, and for moderate temperature increase Δ*T*(*t*), Δ*S*_solvent_(*Q,t*) is a linear function of Δ*T*(*t*), by Δ*S*_solvent_(*Q,t*)=Δ*T*(*t*) × 
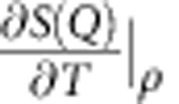
, where 
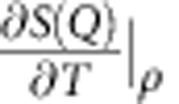
 is the difference scattering signal specific for MeCN at constant density *ρ* (Supplementary Notes 9 and 10; Supplementary Fig. 9). This methodology for analysing Δ*S*_solvent_(*Q,t*) has been developed at third-generation synchrotron facilities^52^,^53^ and has proven a robust and widely applicable approach^32^,^54^,^55^,^56^. In the present analysis, Δ*S*_XDS_(*Q,t*) was independently fitted for each *t* to a linear combination of solute and solvent contributions broadened by the XFEL spectral function (Supplementary Note 8; Supplementary Fig. 8) with *γ*_XDS_ and Δ*T* as free parameters. Figure 5b shows the experimental (black dots) and fitted (purple line) Δ*S*(*Q*) patterns for *t*=25 ps, while Fig. 5c displays the contributions Δ*S*_DFT_(*Q*) (blue line) and 
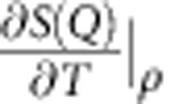
 (red line)^53^ used in the fitting procedure (Supplementary Note 8). While a detailed structural refinement is beyond the signal-to-noise ratio obtainable at the time of the experiment, the two components shown in Fig. 5c represent the major contributions to the observed signal (Supplementary Note 11; Supplementary Fig. 10). Their amplitudes can thus be interpreted as the most significant kinetics of the structural rearrangements in the solute and the solvent. Figure 6a,b contains the experimental *γ*_XDS_(*t*) (blue dots) and Δ*T*(*t*) (red dots) and their respective fits after broadening by the 520±410 fs XFEL IRF (black lines). The step-like increase of *γ*_XDS_(*t*) to 67±4% for *t*>0 matches well the 65±8% obtained for *γ*_XES_ at 20 ps. The temporal evolution of Δ*T*(*t*) follows a single-exponential rise of 12±3 ps and reaches a final value Δ*T*_*f*_=1.0±0.1 K. These XDS measurements therefore capture the structural changes of the solute and the early dynamics of impulsive solvent heating until thermalization. Comparing the timescales extracted independently from TOAS and simultaneous XES–XDS provide the second conclusion reached in this study: the initial charge transfer from the Ru centred to the electronically excited Co centre is 20 times faster than the local thermalization, and the charge localization, along with very large structural rearrangements that occur almost an order of magnitude faster than the thermalization of the hot molecule with its surroundings.

**Figure 7:**
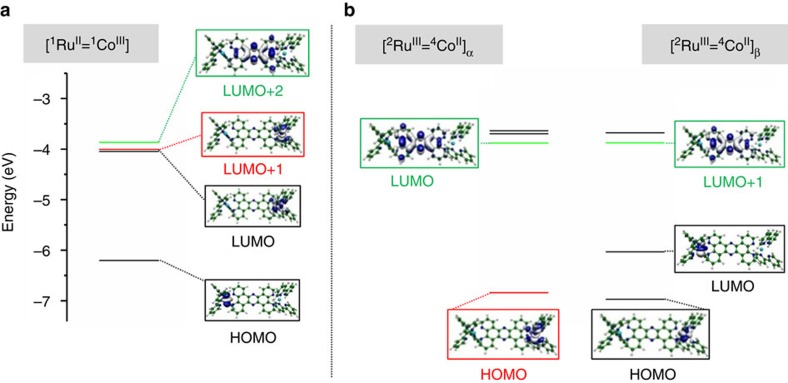
Low-lying electronically excited MOs and frontier MOs. These MOs have been obtained from DFT calculations for (**a**) the LS [^1^Ru^II^=^1^Co^III^] and (**b**) the HS [^2^Ru^III^=^4^Co^II^]_α_ and [^2^Ru^III^=^4^Co^II^]_β_.

By combining ultrafast TOAS, XES and XDS measurements, all the timescales that characterize the non-equilibrated ET within the photoexcited [^1^Ru^II^=^1^Co^III^ (LS)] dyad are now resolved across several length scales, from the population of the optically excited Franck–Condon state to the fully equilibrated charge-separated state. The TOAS and XES experiments track the sub-picosecond electronic dynamics of the ET from the optically bright Ru donor to the optically dark Co acceptor. Three steps are identified on the few picosecond timescale: (1) quasi instantaneous ET from the photoexcited Ru centre to the tppz bridge (*τ*<50 fs), (2) migration through the tpphz bridge (*τ*~0.5 ps) and (3) localization at the Co centre, which undergoes a SST (*τ*~1.9 ps). Contrasting the kinetics observed in the visible and the X-ray spectral range evidences that this last step involves an additional optically dark intermediate, tentatively assigned as the ^2^Co^II^(LS) state. The key role of electronically excited states in promoting such a process is further supported by DFT calculations. The electronic and geometric structures of [^1^Ru^II^=^1^Co^III^ (LS)] and [^2^Ru^III^=^4^Co^II^ (HS)] solvated in MeCN were optimized using DFT. In the latter case of an open-shell system, the unrestricted formalism was applied to obtain the spin-up (α) and spin-down (β) configurations (Supplementary Note 4). From the manifold of virtual unoccupied molecular orbitals (MOs) returned by the DFT calculations, it is clear that the electronic relaxation cascade from the Franck–Condon state should encompass the population of electronically excited species. The virtual MOs of [^1^Ru^II^=^1^Co^III^ (LS)] (Fig. 7a) mirror the frontier orbitals of the fully charge-separated [^2^Ru^III^=^4^Co^II^ (HS)] (Fig. 7b). Specifically, the (HOMO)_α_ of [^2^Ru^III^=^4^Co^II^ (HS)] is very similar to the (LUMO+1) of [^1^Ru^II^=^1^Co^III^ (LS)], while the (LUMO)_α_ and the (LUMO+1)_β_ of [^2^Ru^III^=^4^Co^II^ (HS)] both resemble the bridge-centred (LUMO+2) of [^1^Ru^II^=^1^Co^III^ (LS)]. Confirming the assignment of the intermediate species as the ^2^Co^II^(LS) state, and its role in promoting the ultrafast charge separation in this dyad calls for further investigations with the higher sensitivity and temporal resolution enabled by the ongoing development of timing tools^57^ and self-seeding schemes^58^ at XFEL facilities.

**Figure 8:**
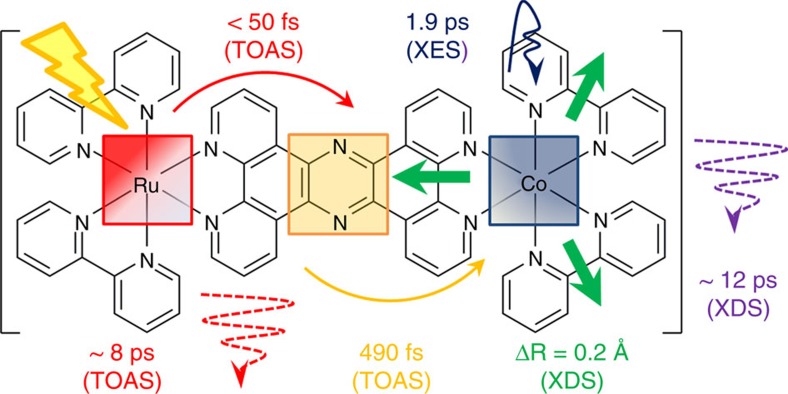
Non-equilibrated ET across the photoexcited [^1^Ru^II^=^1^Co^III^] dyad. The schematic summarizes the fundamental timescales, as obtained from TOAS and combined XES–XDS at the SACLA XFEL facility.

On longer timescales (~10 s of ps) and larger length scales (~10 s of Å), the XDS experiments track the structural changes around the Co centre (Δ*R*~0.2 Å) and the rate of solvent heating (*τ*~12 ps). These simultaneous XES and XDS measurements constitute the first X-ray-based visualization of coupled photoinduced ET and structural rearrangements that both proceed much faster than the local equilibration. The time constant of solvent heating is in good agreement with the one obtained from TOAS (*τ*~8 ps) for the vibrational cooling of the optically bright states (that is, Ru based). The difference between these two rates can be partly ascribed to the existence of hot spots, where some degree of vibrational cooling takes place at the molecular level on the 100 fs timescale, without significant energy transfer to the solvent. Collectively, the ultrafast TOAS, XES and XDS measurements provide direct experimental evidence that non-equilibrated ET mediated by electronically excited molecular states can yield charge-separated species that have undergone profound structural rearrangements through large-amplitude atomic motions, while still competing efficiently with intramolecular vibration energy redistribution and heat loss to the surroundings. The various steps and their respective timescales are illustrated in Fig. 8.

## Discussion

The data quality achieved in this study indicates that these novel analytical X-ray tools are ready to be applied to the diagnostics of fully functional systems. The general implications of the results established in this work are now outlined. Dynamical information very similar to that obtained here is crucially lacking to rationalize the performances of most photoactive donor–acceptor assemblies and to improve the current bottom–up design strategies of photocatalysts. Maximizing the efficiency of photoconversion in supramolecular complexes requires optimizing three seemingly independent steps, namely panchromatic harvesting of sun light (step 1), rapid transfer of the excited electron to the acceptor site (step 2) and stabilization of the charge-separated species (step 3).

This sequence of steps results in a net metal-to-metal ET over large interatomic distances. Extending the absorbance without opening up detrimental loss channels, while promoting the ultrafast electronic localization onto the bridge, constitute intertwined targets in the elaboration of any photoactive dyad^59^,^60^,^61^. From the feedback provided by TOAS about the ET rates, it is possible to tune the excited state manifolds of the donor–bridge units by introducing various electron donating/withdrawing substituents on the peripheral/bridging ligands^61^ to realize concurrently step 1 and 2 for a given molecular architecture. A similar tailoring of the bridge–acceptor units has been hampered so far by the scarcity of techniques capable of probing the non-equilibrated ET dynamics at the catalytic sites, which are usually optically dark centres. This work establishes that measurements at XFEL facilities can now clock the ultrafast electronic localization at the acceptor site. It also unambiguously demonstrates that this ET rate cannot be generally identified with the quenching rate of the bridge-localized states monitored by TOAS, as commonly assumed. Therefore, tracking ET rates directly with element- and spin sensitivity should greatly contribute to rationalizing step 2 and the associated trends in structure–activity relationships. Advanced modelling with DFT, time-dependent DFT and QM/MM MD^62^ will have to be extensively employed to assist the mechanistic interpretations of the observations. Preliminary QM/MM (solute/solvent) equilibrium MD simulations of [^1^Ru^II^=^1^Co^III^ (LS)] and [^2^Ru^III^=^4^Co^II^ (HS)] can already confirm the long-time-simulated structural change around the Co centre reported in this communication (Supplementary Note 12; Supplementary Fig. 11). Such theoretical guidelines will prove essential, since the predominant participation of highly excited states necessitates going beyond the frame of Marcus theory. For example, no clear-cut argumentation based on the driving force, reorganization energy or electronic coupling can yet be put forward to explain why the charge separation in [^1^Ru^II^=^1^Co^III^ (LS)] is an order of magnitude faster than in closely related complexes (for example, [^1^Ru^II^=^1^Os^III^ (LS)]^35^. The present experiments also reveal that the ET and the SST proceed much faster than the dissipation of excess energy to the environment. Moreover, the energy-storing ET step Ru→Co occurs on the same timescale as the intramolecular redistribution of vibrational energy, which is a prerequisite for full photon energy utilization. In other words, step 2 has the potential to produce ‘hot’ activated acceptors that can drive highly endoenergetic reactions. The systematic exploitation of this aspect will require extensive input from ultrafast X-ray experiments whenever the active site is optically dark. By merging the information obtained from ultrafast optical and X-ray experiments, it becomes possible to follow vectorial non-equilibrated ET throughout a dynamically evolving molecular architecture, as it interacts with the immediate surroundings. Optimizing the sequence of step 1, 2 and 3 can now be approached as a single-integrated task^63^,^64^.

In conclusion, the two emerging femtosecond X-ray measurement techniques XES and XDS have been combined at the SACLA XFEL facility to unveil the fundamental timescales of non-equilibrated ET in a bimetallic donor–acceptor complex. XES follows the ultrafast reduction of the Co centre and the accompanying spin-state transition that takes only about 2 ps. Correlation with optical measurements demonstrates the participation of at least one optically dark step. XDS catches the structural changes of the solute, as well as the onset of the structural response arising from ultrafast solute-mediated solvent heating. The stabilization of the charge-separated state over 13 Å is determined to be faster than the large structural reorganization of the complex and dissipation of excess energy in the surroundings. Insights provided by DFT calculations suggest that the ultrafast ET is promoted by electronically excited molecular states, a key mechanistic feature of this non-adiabatic process. Ascertaining the mechanisms of non-equilibrated ET in the homogeneous phase is paving the way to controlling and manipulating hot ET, which is generally foreseen as one of the routes to follow if we are to match the unrivalled efficiency of natural photosynthesis.

## Methods

### Materials

The dyad studied in this work is the dinuclear complex [(bpy)_2_Ru^II^(tpphz)^1^Co^III^(bpy)_2_]^5+^ (abbreviated as [Ru^II^=^1^Co^III^]) where ‘bpy’ is 2,2′-bipyridine, and ‘tpphz’ tetrapyrido[3,2-*a*:2′,3′-*c*:3′′,2′′-*h*:2′′:2′′′-3′′′-*j*]phenazine. Its PF_6_ salt was synthesized following the improved protocol given in ref. 34.

### Femtosecond transient absorption spectroscopy

The femtosecond laser setup used for transient absorption spectroscopy has been previously described^65^. The excitation beam was depolarized and set to 400 nm. The fitting procedure is described in Supplementary Note 1 with Supplementary Fig. 1.

### Experimental X-ray setup

An optical pump-X-ray probe setup combining XES and XDS was implemented at beamline BL 3 of the SACLA XFEL facility, Japan (Fig. 1a). A 6 mM solution of [^1^Ru^II^=^1^Co^III^] in acetonitrile (MeCN) was continuously circulated in a free-flowing planar liquid sheet (100 μm). The molecules were optically excited at 400 nm (60 fs pulse length, 500 μm FWHM focus spot and 220 μJ per pulse). The probe consisted of 8 keV X-ray pulses (10 fs pulse length, 0.3% bw, 450 μm FWHM beam size and 10^10^ photons per pulse) generated by SACLA. Both laser and X-rays were operated at a 10 Hz repetition frequency. The two beams crossed under a 10° angle in the horizontal plane. The effective time resolution was given by the shot-to-shot jitter between the optical and XFEL pulses.

A 4″ diameter spherically bent Si(531) analyser crystal (1,010 mm bent radius) working in the Rowland circle geometry was used to measure the Co Kα_1_ emission maximum at 6.93 keV. It was placed at a scattering angle of 130°, to resolve and focus the X-ray emission at a Bragg angle of 77° in the horizontal plane. This signal was detected by a multiport-charged-coupled device (MPCCD) area detector. Rotation of the analyser crystal with concurrent movement of the MPCCD allowed for energy (wavelength) selection. The XDS signal was recorded on a second MPCCD detector placed 3.5 cm behind the sample (with the direct beam blocked), allowing for detection in a *Q*-range spanning from 0.4 to 3.2 Å^−1^. The XES and XDS detectors were read out after every X-ray pulse. The signal of interest was extracted from a differential measurement with and without laser irradiation on the sample, that is, as a [laser_ON_ (*t*)−laser_OFF_] traces. Two types of scans (kinetic scans and energy scanes) were acquired. For the kinetic scans, the time delay *t* between the laser and the X-ray pulse was varied by steps of 0.3 (for XES measurements), 3 (for combined XES/XDS measurements) or 10 (for combined XES/XDS measurements). The signal was integrated for 20 s (corresponding to 200 X-ray pulses, with the detector signal read out for each individual pulse). For the scans with a 0.3 ps step size, negative delays of −100 ps (corresponding to the X-rays arriving 100 ps before the laser pump) were interspaced such that every fourth delay was a laser_OFF_ measurement allowing for correction of any long-term drifts in the signal during the acquisition.

For the energy scans of the XES intensity at constant *t*, the signal was integrated for 4 s (corresponding to 40 X-ray pulses) at each energy point.

### XES data analysis

The fine structure of the K_α_ spectra originates from multiplet and spin orbit interactions. In transition metal systems, they are highly sensitive to the oxidation state and to the number of unpaired electrons^24^,^26^,^66^. The two-dimensional images containing the XES signal were recorded by the MPCCD detector at the focus of the analyser crystal. The images were corrected, and the photons of each exposure were explicitly counted for every X-ray pulse as described in Supplementary Note 2. The Kα_1_ spectra were constructed from the energy scans, resulting in the transient XES signal Δ*S*_XES_(*t*) curves shown in Fig. 2a (main text). A lineshape analysis is typically applied to extract the charge and spin information from such XES data^26^,^67^,^68^,^69^. However, the short effective data collection time of the experiments reported in this work did not permit to obtain a sufficiently large set of spectra with known references and good statistics to fully exploit this approach. It was nevertheless indirectly applied to follow the spectral variations, since upon increase in spin state^26^, the Kα_1_ spectra of Co undergoes a clear broadening. As such, the linewidth in Kα_1_ spectra can also be used to calibrate the charge and spin momentum *S* on the Co centre. For the data presented in Fig. 4a, the difference in linewidth between the ground state and the photoexcited state is 0.6 eV. This value corresponds to a spin-state change Δ*S* of 1.5. Since *S=0* in the initial ^1^Co^III^ state, the final spin state is *S*=3/2, that is, that of a HS ^4^Co^II^ (Supplementary Fig. 4). Since the total Kα_1_ emission intensity does not depend on the charge and spin state, the relative changes in the width of the emission line for the different Co species directly result in an inverse lowering of the maximum emission intensity, that is, the peak height. This is the parameter measured and plotted in the kinetic traces presented in Fig. 4b and Supplementary Fig. 5.

### Kinetic models for the XES data

Two kinetic models of charge and spin dynamics have been tested against the observed XES kinetics. In model A, concerted ET and SST ^1^Co^III^(LS)→^4^Co^II^(HS) take place with an overall rate constant 1/*τ*_0_, where *τ*_0_=(490 fs)^−1^ is the lifetime of the reduced bridge as measured with TOAS. Starting with an initial excitation fraction of *γ*_0_, the time-dependent concentrations of the transient species for this model A is:


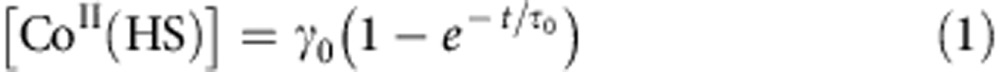


In model B, sequential ET ^1^Co^III^ (LS)→^2^Co^II^(LS) with a rate constant 1/*τ*_0_ matching the decay rate of reduced bridge from TOAS is followed by the SST ^2^Co^II^(LS)→^4^Co^II^(HS) with a free rate constant 1/*τ*_1_, resulting in ^1^Co^III^ (LS)→^2^Co^II^(LS)→^4^Co^II^(HS)Starting with an initial excitation fraction of *γ*_*0*_, the time-dependent concentrations of the transient species for this model are:


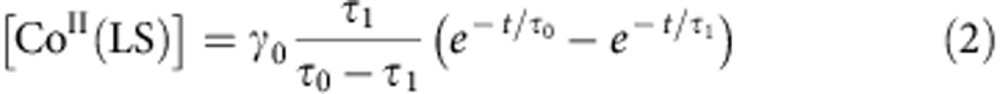



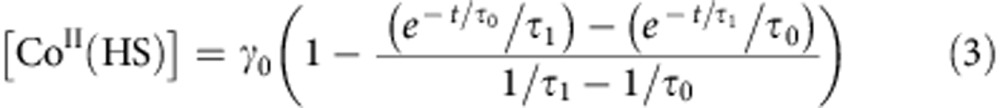


The thermally activated back ET to the ground state was omitted since the lifetime of the charge-separated [^2^Ru^III^=^4^Co^II^(HS)] is 45 ns^35^, that is, three orders of magnitude longer than the temporal window studied in this work. The time evolution of the XES signals was then modelled by convoluting the transient concentrations introduced above with a Gaussian IRF. Taking model A as an example, the resulting kinetics were given by:


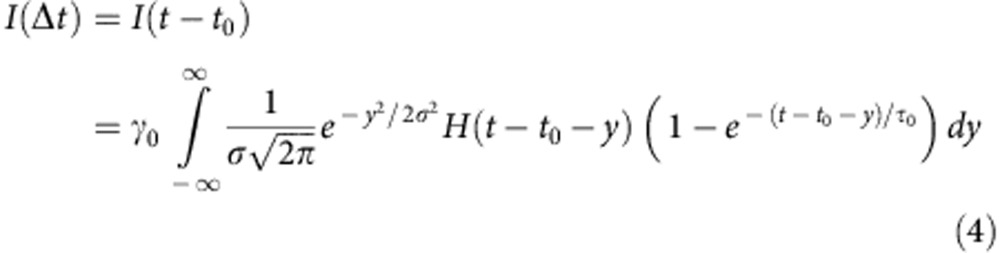


where *σ* is the width of the Gaussian broadening, *t*_0_ is time 0, *γ*_0_ is the amplitude of the difference signal at *t*_0_ and *H* is the Heaviside step function.

The corrected Akaike information criterion (AICc) is then used to compare the relative goodness of the fit weighted by the model complexity^70^. Briefly, the AIC is introduced as:


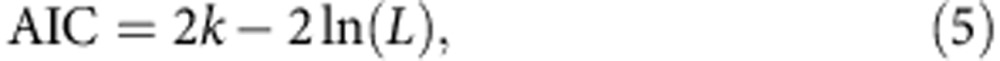


where *k* is the number of fit parameters and *L* is the maximized likelihood value of the fit defined as:


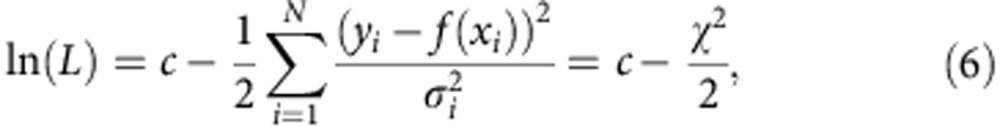


where *N* is the number of data points, *y*_*i*_ is the measured value, *f(x*_*i*_) is the fit value, *σ*_*i*_ is the standard deviation of the measured values and *c* is a constant. It has been shown that, for small data sets, the bias-corrected AIC value (AICc) should be used for model comparison:


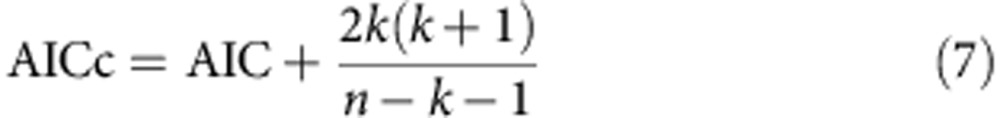


The direct relation of the AIC value to the maximized likelihood entails that it can be directly determined from the reduced *χ*^2^:


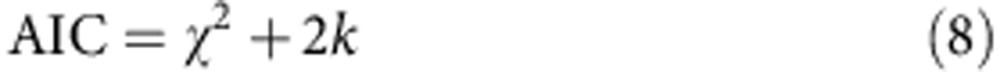


Considering two fitting models (1 and 2) of AICc values AICc_1_ and AICc_2_, the probability *p* that model 1 is the most likely of the two models is given by:


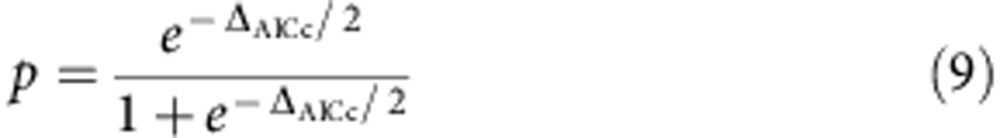


where Δ_AICc_=AICc_1_−AICc_2_.

The best fit parameters for the two models introduced above are listed in Table 1, along with their AICc-derived probabilities *p*_A_ and *p*_B_, taking B as reference. Considering these probabilities, there is substantial evidence for rejecting A in favour of B. The Supplementary Fig. 5 shows the fits resulting from models A (cyan) and B (blue) against the XES data. Model A systematically underestimates the data in both the region around *t*_0_, as well as the region between 2 and 6 ps.

### XDS data analysis

Two-dimensional X-ray scattering images were recorded by the MPCCD detector behind the sample. These images were corrected (for sample absorption, solid angle coverage and detector efficiency as a function of angle and polarization), azimuthally integrated and individually scaled to yield one-dimensional radial curves *S*(*Q,t*), which were used to construct the difference X-ray scattering signals Δ*S*_XDS_(*Q,t*)*=*[*S*_ON_(*Q,t*)—*S*_OFF_(*Q*)] for each time delay *t*, as described in detail in Supplementary Note 5. The set of Δ*S*_XDS_(*Q,t*) was subsequently analysed in a framework developed for treating such data containing a significant contribution from a fluctuating background. The method relies on fitting SVD-detected background components simultaneously with components calculated form a physical model (see main text) to Δ*S*_XDS_(*Q,t*) for each *t*. This approach, described in detail in ref. 51, is summarized as applied to the present data sets in Supplementary Notes 6 and 8.

## Additional information

**How to cite this article:** Canton, S. E. *et al.* Visualizing the non-equilibrium dynamics of photoinduced intramolecular electron transfer with femtosecond X-ray pulses. *Nat. Commun.* 6:6359 doi: 10.1038/ncomms7359 (2015).

## Supplementary Material

Supplementary Information Supplementary Figures 1-11, Supplementary Notes 1-12 and Supplementary References

## Figures and Tables

**Table 1:** Analysis of the transient XES signal.

**Model**	***t***_**0**_ **(ps)**	***γ***_**0**_ **(ps)**	***τ***_**1**_ **(ps)**	***σ*****(ps)**	***P***
A	1.5±0.3	0.66±0.02	—	1.5±0.3	0.04
B	0.05±0.13	0.67±0.02	1.9±0.5	0.52±0.41	0.50

The fitting parameters were *t*_0_ (time 0), *γ*_0_ (initial excitation fraction), *τ*_1_ (free rate constant) and *σ* (width of the Gaussian broadening) for models A and B. The corresponding *p* parameters are given in the last column.
