# Supplementary material for: Visualizing the non-equilibrium dynamics of photoinduced intramolecular electron transfer with femtosecond X-ray pulses
Source: Nat Commun. 2015 Mar 2;6:6359. doi: 10.1038/ncomms7359 (PMC4366532; doi:10.1038/ncomms7359)
Supplement: Supplementary Information — Supplementary Figures 1-11, Supplementary Notes 1-12 and Supplementary References [file ncomms7359-s1.pdf]

Supplementary Figure 1: TOAS data analysis fit

2-component fit

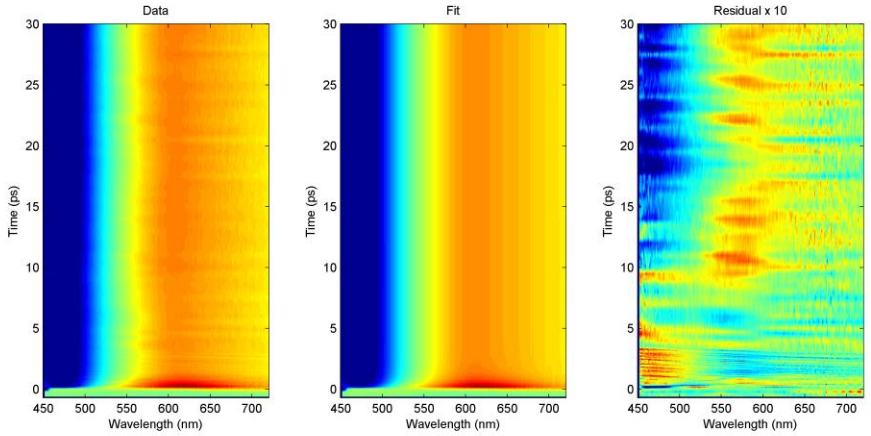

3-component fit

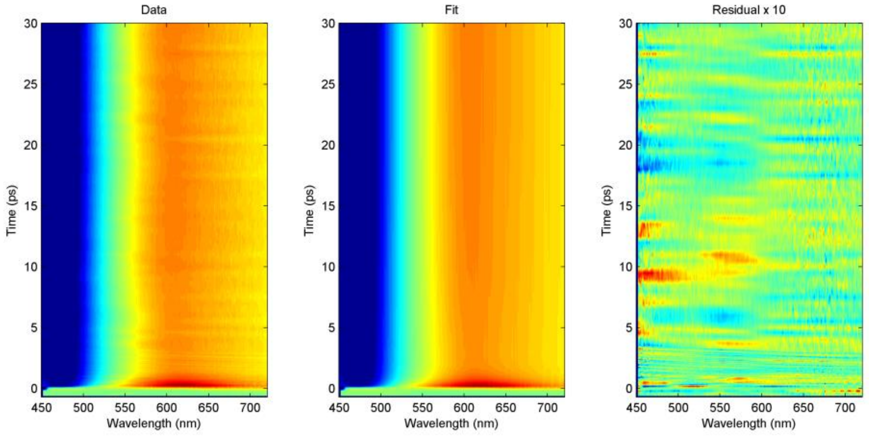

Supplementary Figure 1: TOAS data, fit and residual from a global analysis using either 2 or 3 exponential components.

Supplementary Figure 2: TOAS data analysis results

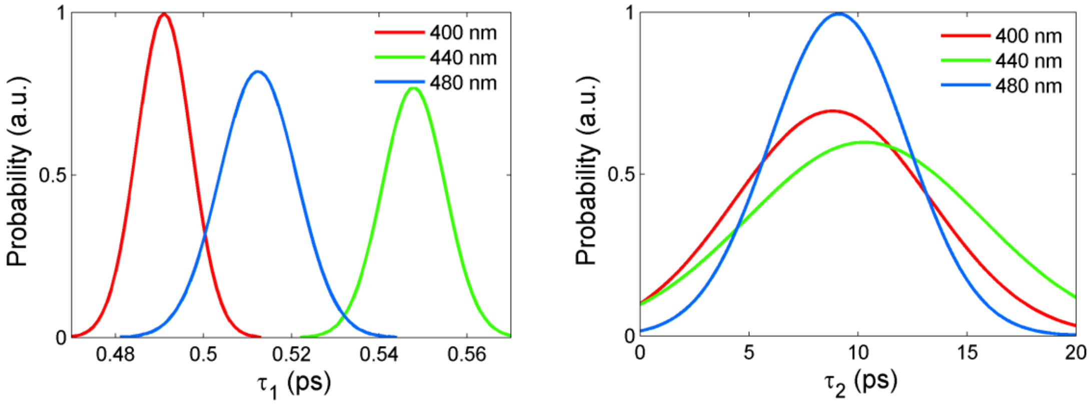

Supplementary Figure 2: Probability distributions of the time constants τ_1 (left) and τ_2 (right) extracted from the global analysis of data recorded at different excitation wavelengths.

Supplementary Figure 3: XES data extraction and reduction

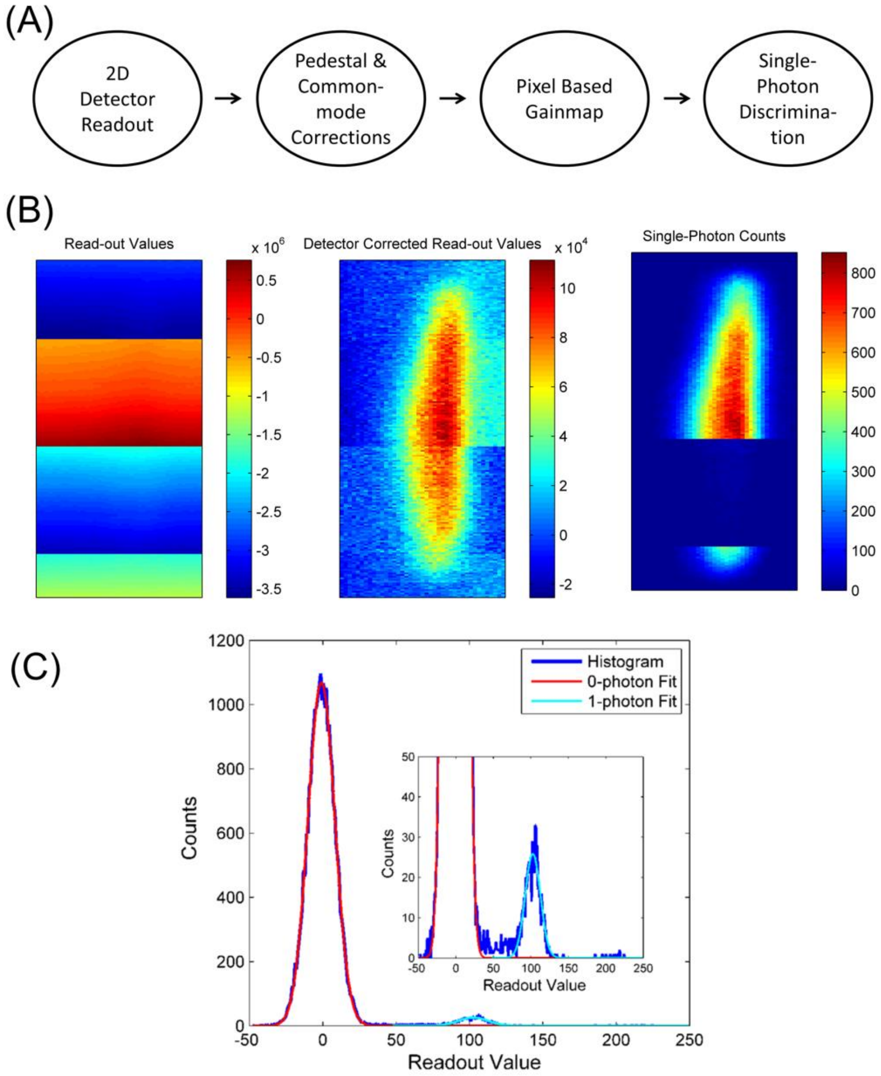

Supplementary Figure 3: (A) Flowchart of the XES data extraction-correction process. (B) Processing of an XES kinetic-scan: (Left) Sum of the read-out values (ROVs) for each pixel after read-out (step 1), (Middle) Summed ROVs after detector corrections, (step 2), (Right) Summed photon counting events for each pixel after single-photon discrimination (step 3). Some of the data had to be discarded due to high read-out noise, resulting in the empty section on the right panel. (C) Single-pixel histogram of a strongly illuminated pixel after step 1 and 2. The peak at ROV = 0 corresponds to readout of the pixel after no photons were detected, the peak at ROV = 100 corresponds to 1-photon counting events, while the clustering of counts observed around ROV = 210 originates from 2-photon events.

Supplementary Figure 4: XES r□XESleme□s□XESle

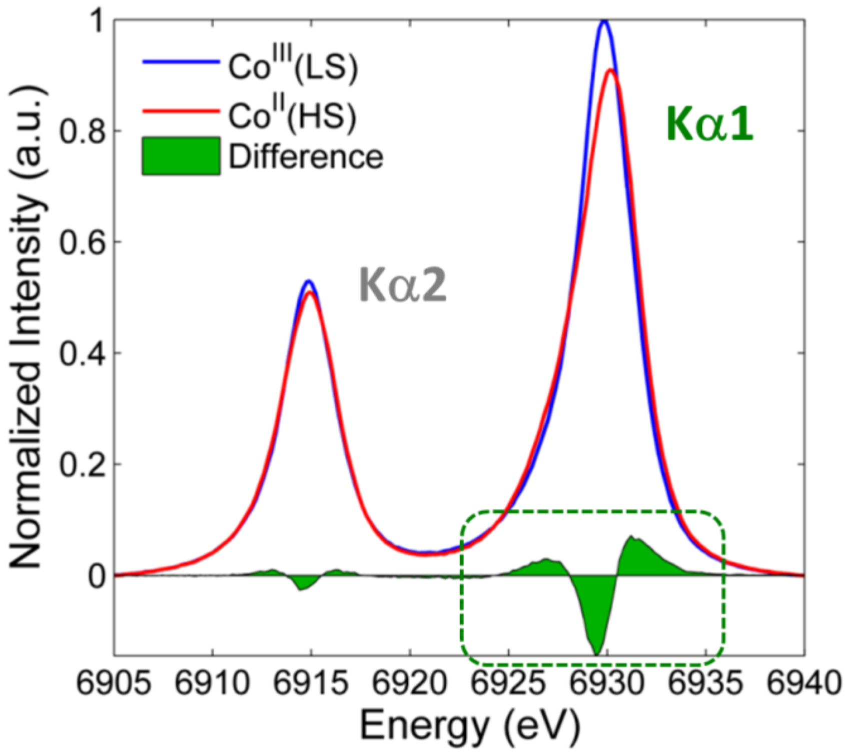

Supplementary Figure 4: Co Kα_1 and Kα_2 XES lines for a Co^{III}(LS) sample (i.e. [Co(bpy)$_3$(PF)$_6$]) (blue trace) and a Co^{II}(HS) sample (i.e. [Co(bpy)$_2$(NCS)$_2$]) (red trace). The difference (green trace in dashed box) is the reference difference signal shown in Figure 2A.

Supplementary Figure 5: Comparison between XES fitting models

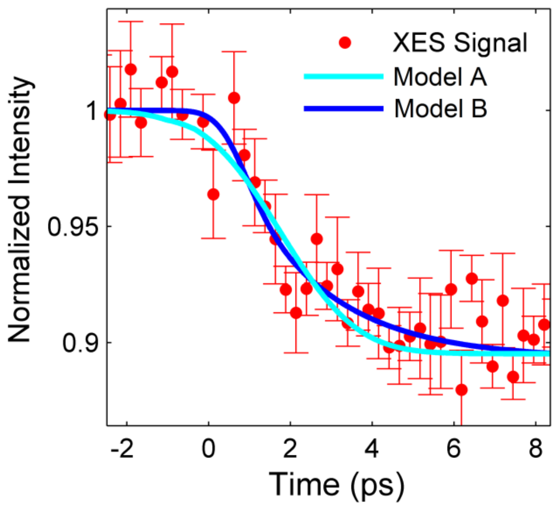

Supplementary Figure 5: The resulting fits to the data of models A (cyan) and B (blue).

Supplementary Figure 6: SVD-based background-removal of XDS data.

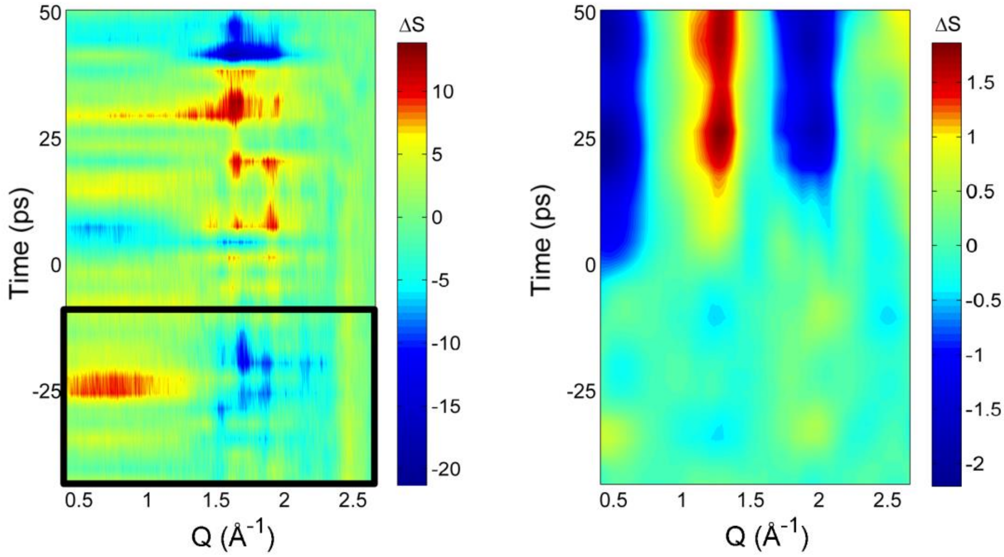

Supplementary Figure 6: $\Delta S_{XDS}(Q,t)$ data before (left) and after (right) the SVD-based background-removal procedure. The black box on the left panel shows the subset of data used to construct the SVD components.

Supplementary Figure 7: XDS sample reference signal

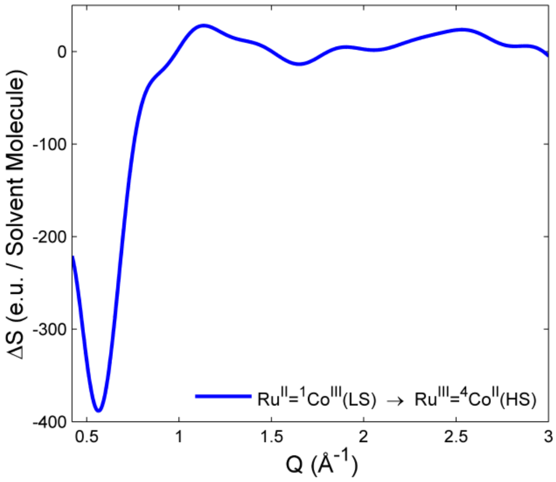

Supplementary Figure 7: $\Delta S_{solute}(Q)$ resulting from a [^{1}Ru^{II}=^{1}Co^{III}(LS)] to [^{2}Ru^{III}=^{4}Co^{II}(HS)] conversion.

Supplementary Figure 8: XDS solvent reference signal

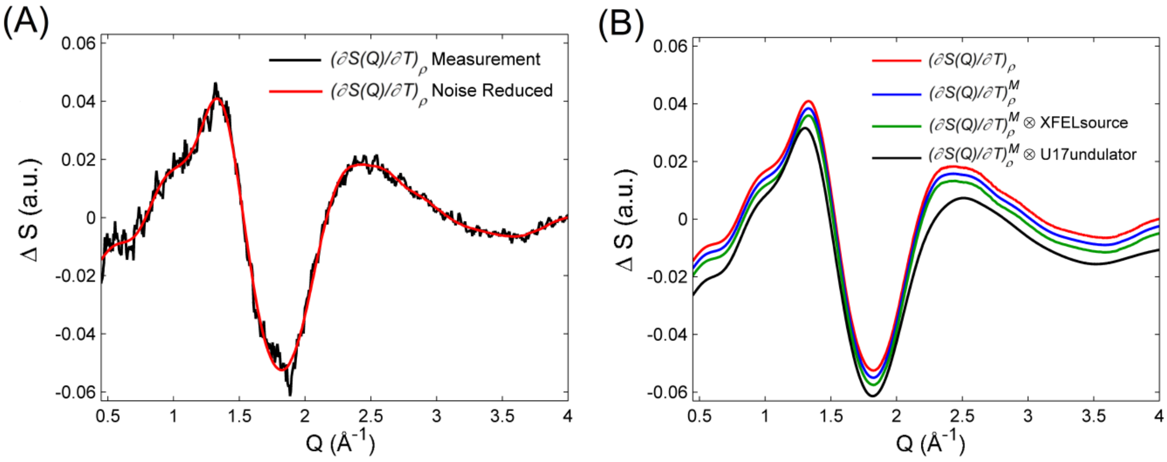

Supplementary Figure 8: The effect of the X-ray source energy spectrum on the temperature solvent differential (TSD). (A) Experimental TSD obtained at ID09b (black) and noise-reduced profile (red). (B) A "monochromatic" TSD (blue) was obtained by deconvoluting the noise-reduced profile (red) by the energy spectrum of the ID09 multilayer optics. This simulated "monochromatic" curve was then convoluted with the energy spectrum of the XFEL source (green) and with the U17 pink beam at ID09 (black) for comparion. The traces are vertically offset for clarity.

Supplementary Figure 9: Pairwise radial distribution functions of acetonitrile

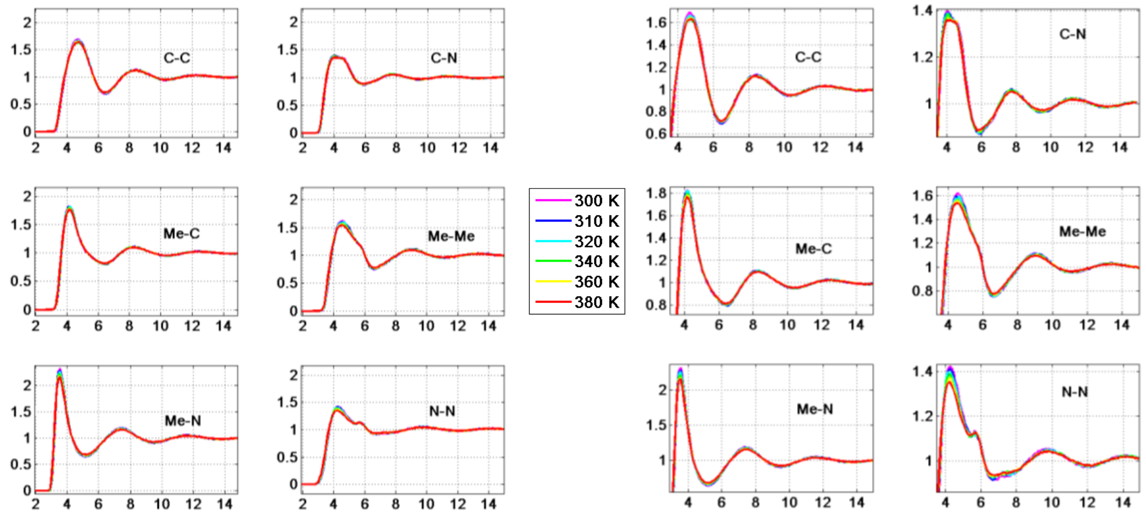

Supplementary Figure 9: Pairwise radial distribution functions o for the constituent components of acetonitrile.

Supplementary Figure 10: Dependency of the simulated XDS signal on the Co-N bond length elongation

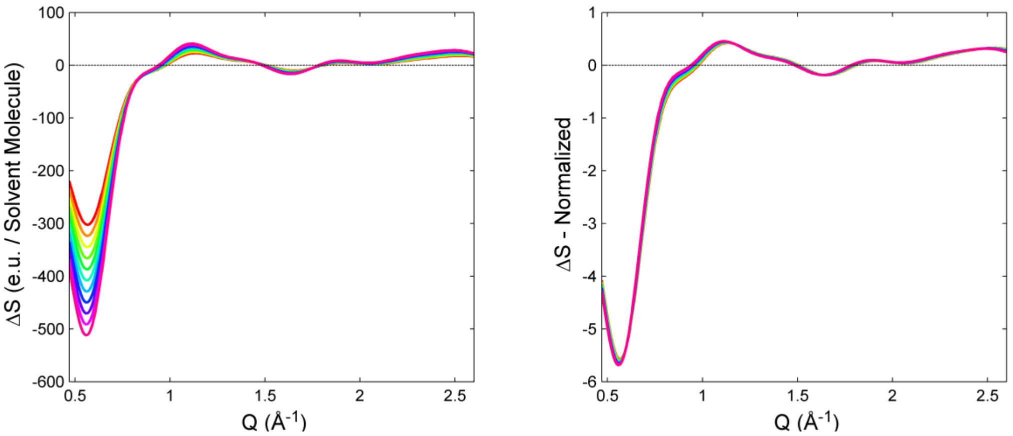

Supplementary Figure 10: Simulated $\Delta S_{XDS}(Q)$ curves for the structural transition from $[^{1}Ru^{II}{=}^{1}Co^{III}]$ to $[^{2}Ru^{III}{=}^{4}Co^{II}]$, where the Co-N bond length elongation – ΔR(Co-N) – has been varied between 0.15Å and 0.25 Å. The left panel shows the curves on an absolute scale, the right panel shows the same data normalized to the signal amplitude.

Supplementary Figure 11: Dependency of the simulated XDS signal on the Co-N bond length elongation

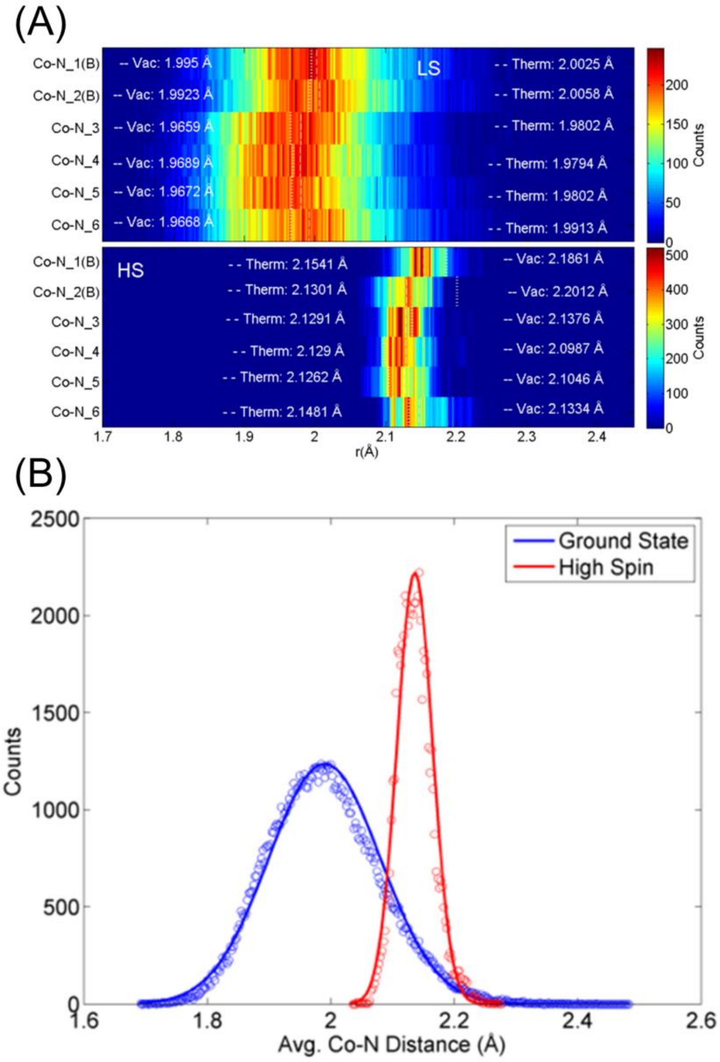

Supplementary Figure 11: (A). Thermal distribution of individual Co-N bond lengths for (a) the [$^1Ru^{II}$=$^1Co^{III}$(LS)] ground state and (b) the [$^2Ru^{III}$=$^4Co^{II}$(HS)] charge separated state, where the Co^{II} center is in the HS state. (B) Thermal distribution of average Co-N bond lengths for the [$^1Ru^{II}$=$^1Co^{III}$(LS)] ground state and the [$^2Ru^{III}$=$^4Co^{II}$(HS)] charge separated state, where the Co^{II} center is in the HS state.

Supplementary Note 1: TOAS data analysis

The TOAS data were fitted within a standard global analysis framework.[1] The number of required decay associated spectra (DAS) can be estimated by inspection of the residuals, as shown in Supplementary Figure 1.

With a two-component fit, the best results are obtained with one sub picosecond and one nanosecond component (i.e. without the vibrational cooling). There is clear structure in the residual of these two-component fit showing that a three-component model (and thus the effects of vibrational cooling) is required to describe the data. The derived time constants are all dependent on the excitation wavelength. This is illustrated for three different excitation wavelengths (400, 440 and 480 nm) in Supplementary Figure 2.

Supplementary Note 2: XES data extraction and reduction

The low readout noise of the MPCCD detector allowed explicit single-photon discrimination of the XES signal. A flow-chart of the data extraction-correction process is shown in Supplementary Figure 3A.

<u>Step 1: Readout</u>

The MPCCD detector is read out as a 2D image containing the read-out value (ROV) of each pixel.

<u>Step 2: Pedestal and common-mode detector corrections</u>

The so-called “pedestal” correction ensures that the zero-photon ROVs of all the pixels are close to 0 analogue-to-digital units (ADU). A set of 15000 dark-measurements (i.e. images with the X-rays shutter closed and no X-rays hitting the detector) was averaged,

yielding the average 0-photon signal level for each pixel. This background was subtracted from all further images. Three sets of such dark-measurements were recorded in the course of the experimental run in order to confirm that this background did not change over time. Common-mode artefacts arising from amplifier/current supply electronics were negligible.

Step 3: Calibration and scaling (pixel-based gainmap and single photon discrimination)

A pixel-specific gainmap constructed from single-pixel ROVs was applied. A typical histogram of the ROVs for a pixel seeing seeing a high photon intensity is plotted in Supplementary Figure 3C (blue line), the inset shows a zoom-in.

The peaks centered at ROV= 0 ADU, at ROV= 100 ADU and the clustering of counts around 210 ADU correspond to 0-, 1- and 2-photon counting events respectively. For each X-ray illuminated pixel, Gaussian functions of RMS σ were fitted to the 0-photon signal (red line), and the 1-photon signals (cyan line) individually. The single-pixel gainmap was constructed by defining the center peak positions of the two Gaussians to 0 and 1 respectively. As can be seen from the inset in Supplementary Figure 3C, many ROVs fall between the 0- and 1-photon peaks. This interval is assigned to fractional photon events. The most robust approach to account for them was to define the number of detected 1-photon and 2-photon events in an exposed image as the number of pixels for which the single-pixel gain-corrected ROVs were such that $0.5<ROV<1.5$ and $ROV>1.5$ respectively. Since the maximum count rate of any pixel was ~0.025 ph/exposure, gain-corrected ROVs corresponding to 3 or more photons were not considered. A lower threshold of 9σ of the zero-photon readout was enforced in the single-photon discrimination to remove false counting events from the readout noise. Supplementary Figure 3B follows a detector image across the 3 stages of the data extraction-correction procedure. Note that the high read-out noise of the third detector from the top made explicit single-photon counting in this element impossible, resulting in the absence of data in the corresponding panel of Supplementary Figure 3B. Even though this approach discards all

photons hitting 1/3 of active area of the detector, it resulted in the best signal to noise ratio, with the mean standard deviation of each data point typically being within 30% of that expected from a true Poisson distribution. The final signal strength obtained in the Co Kα_1 emission peak is the sum of all the detector counts for a given pulse and was typically ~20 photons/pulse, while the background count was ~0.2 photons/pulse.

Supplementary Note 3: XES data analysis

The Kα_1 spectra originate from multiplet and spin orbit interactions. In transition metal systems, it is highly sensitive to the oxidation state and to the number of unpaired electrons. A frequent evaluation approach that is applicable to the interpretation of photoinduced transient Kα 2p$\rightarrow$1s and Kβ 3p$\rightarrow$1s spectra is based on constructing differences of steady-state spectra from suitable reference complexes; the integrals of the absolute values of the difference spectra are then proportional to the conversion yield. This approach is somewhat similar to the treatment of X-ray dichroism, although sum rules do not apply, and one needs to find acceptable reference compounds.[2] Still, this approach is quantitative for two-state transitions,[3] and can be indicative of more complex spin-state transitions, as demonstrated in numerous studies[4,5,6,7,8,9,10,11,12]

However, in the experiments reported in this work, the short effective data collection time did not permit to obtain a sufficiently large set of spectra with clear references and good statistics to fully exploit this approach. It was nevertheless indirectly applied to follow the spectral variations, since for the Kα_1 spectra, the linewidth (full-width at half-maximum FWHM) can also be exploited to calibrate the spin momentum on the cobalt center. Although the 2p-3d exchange interaction is rather small, its variation upon increase in spin-state appears as a clear broadening[2]. After a careful comparison with the static lineshapes

obtained at the ID26 beamline of the ESRF (shown in Supplementary Figure 4), we can determine that the 0.6 eV FWHM difference observed in the current experiment for the ground and photoexcited state corresponds to a spin-state change of $\Delta S=1.5$ at t=20 ps. Since the initial spin momentum of the $^{1}Co^{III}$ is $S=0$, this corresponds to $S=3/2$, a spin-state HS of $^{4}Co^{II}$. Given that the total integrated $K\alpha_1$ emission intensity does not depend upon the charge and spin-state[2] the relative changes in the FWHM of the emission line for the different Co species directly result in an inverse lowering of the maximum emission intensity, i.e. the peak height. This is the parameter measured and plotted in the kinetic traces presented in the main text.

Supplementary Note 4: Computational details for the DFT optimization

All the calculations were carried out with the ORCA program package.[13,14] The geometries of $[^{1}Ru^{III}(=)^{1}Co^{III}$ (LS)], $[^{2}Ru^{III}(=)^{4}Co^{II}$ (HS)$]_\alpha$ and $[^{2}Ru^{III}(=)^{4}Co^{II}$ (HS)$]_\beta$ were fully optimized with the B3LYP*[15]/TZVP method. This functional has provided reasonable results for the structures and energetics of the LS and HS states of transition metal complexes.[16,17,18,19,20] The conducting-like screening solvation model (COSMO)[21] was applied with ε = 36.6 as appropriate dielectric constant for acetonitrile. The Kohn-Sham orbitals were extracted from the converged wave functions corresponding to single point calculations performed at the optimized geometries.

Supplementary Note 5: XDS Data Analysis - Construction of the XDS difference signals, *$\Delta S_{XDS}(Q,t)$*

The 2D X-ray Diffuse Scattering (XDS) images extracted from the MPCCD detector were corrected to account for the effects of:

1) the X-ray beam polarization,

2) the X-ray absorption of 8 keV photons throughout the liquid sheet ,

3) the solid angle subtended by each detector pixel,

4) the X-ray absorption probability of an 8 keV photon within a pixel.

The resulting images were then integrated azimuthally around the beam center (found by circle-fits to the liquid-peak in the 2D images) producing 1D curves of the scattering intensity $S(Q)$, where $Q = \frac{4\pi \sin(2\theta)}{\lambda}$ is the momentum transfer, 2θ is the scattering angle, and λ is the X-ray wavelength. The first step in the analysis of $S(Q)$ was a simple filtering procedure. Any exposure where either I_0 (the incident intensity) or I (the integrated intensity on the MPCCD) fell outside one standard deviation from the mean of either of the two intensities was discarded. The remaining $S_{XDS}(Q,t)$ were averaged for each time-delay and scaled to a simulated scattering curve $S_{Sim}(Q) = S_{Coh}(Q) + S_{Incoh}(Q)$, which is the sum of the coherent $S_{Coh}(Q)$ and incoherent $S_{Incoh}(Q)$ scattering arising from a "Liquid Unit Cell", L.U.C, i.e. an ensemble of molecules representing the stoichiometry of the sample. In the present case, the L.U.C consisted of a single Ru^{II}=$^1Co^{III}$ molecule, 5 PF_6^- ions and 19.15 M/6 mM = 3191 acetonitrile molecules. $S_{Coh}(Q)$ was calculated from the orientation-averaged Debye equation by using the isolated-atom formalism. The atomic form factors were described by the Cromer-Mann parameterization[22]. $S_{Incoh}(Q)$ was obtained from the parameterization provided by Hajdu[23]. Scaling $S(Q,t)$ to the L.U.C. $S_{sim}(Q)$ was done by minimizing [$S_{Sim}(Q) - \alpha \cdot S_{XDS}(Q,t)$] for 1.5 $Å^{-1}$ <Q< 1.8 $Å^{-1}$ around a nodal point in the difference scattering signal[24]. This step effectively put the $S_{XDS}(Q,t)$ on an absolute scale of electron units/LUC[25]. The XDS difference

signals $\Delta S_{XDS}(Q,t)$ were calculated for each t by subtracting the average signal recorded for the unpumped sample $\Delta S_{XDS}(Q,t) = S_{XDS}(Q,t) - S_{XDS}(Q)_{Off}$, where $S_{XDS}(Q)_{Off}$ is defined as the mean of the earliest 12 time-delays for which the X-ray pulses arrived from 12 ps to 45 ps before the laser pulse, namely $S_{XDS}(Q)_{Off} = \sum_{i=1}^{11} S_{XDS}(Q,t_i) / 12$

Supplementary Note 6: XDS Data Analysis - SVD-based background contributions to $\Delta S_{XDS}(Q,t)$

The XDS difference signals $\Delta S_{XDS}(Q,t)$ obtained for $t<0$ contain a considerable amount of noise. If the background contributions were constant during acquisition, these $\Delta S_{XDS}(Q,t)$ should in principle amount to statistical fluctuations. In order to identify and remove these contributions, Singular Value Decomposition (SVD) was applied to the set of $\Delta S_{XDS}(Q,t)$ curves calculated in the region used to construct $S_{XDS}(Q)_{Off}$. In general, SVD analysis decomposes any matrix X as the product $X = U*S*V^T$, where U and V are unitary matrices, while S is a diagonal matrix. Taking X as the data matrix, the columns of U are the singular vectors representing the variation in the data matrix X along the time coordinate, and V contains the time-dependency of this variation. The elements of the diagonal matrix S denote the relative magnitudes of the singular vectors. Inspection of S indicates that five singular vectors are sufficient to accurately represent the variation in the $S_{XDS}(Q)_{Off}$ data matrix, as the singular values S_{ii} for i > 5 become very small. The residual signal $\Delta S_{XDS}(Q, t)_{res} = |\Delta S_{XDS}(Q,t) - \Sigma\alpha_i \cdot S_{ii} \cdot U_{ji}|$ for each t as a function of the 5 scaling parameters $\alpha_{1\text{-}5}$ is shown in Supplementary Figure 6. A 10 point smoothing was applied to the individual traces, followed

by a 3x3 point median filtering. For all time delays, the fluctuating background can therefore be accurately described by a sum of five singular vectors such that $\Delta S_{XDS}(Q,t)_{back}$ = $\sum_{i=1-5}\alpha_i(t)\Delta S_i^{SVD}(Q)$, where $\Delta S_i^{SVD}(Q) = S_{ii}\cdot U_{ij}$. Observing that this background-removal procedure reduced the subset of data for t < 0 to a negligible level validated the use of the SVD-determined descriptor vectors in an unconstrained fit of the $\Delta S_{XDS}(Q,t)$. The implementation of this methodology in the full global-fit analysis is described in the next section.

Supplementary Note 7: XDS Data Analysis - Sample contributions to $\Delta S_{XDS}(Q,t)$

The $\Delta S_{XDS}(Q,t)$ contains contributions from all the structural changes that occur in the probed sample volume upon laser excitation. It is usually expressed as the sum of 3 terms:

i) The solute term $\Delta S_{solute}(Q,t)$, arising from structural changes in the solute

ii) The solvent term $\Delta S_{solvent}(Q,t)$, arising from structural changes of the bulk solvent

iii) The solute-solvent cross-term, arising from structural changes of the solvent-shell surrounding the solute.

<u>The solute term</u>

This term was estimated from the molecular geometries of the [$^1Ru^{II}$=$^1Co^{III}$(LS)] and [$^2Ru^{III}$=$^4Co^{II}$(HS)] optimized through DFT using the ORCA 2.8 program package[13] with the BP86[26,27] gradient-corrected (GGA) exchange-correlation functional and Gaussian type TZVP basis set. The conducting-like screening solvation model (COSMO)[21] was applied with ε =

36.6 as appropriate dielectric constant for acetonitrile. The coherent scattering of these two structures was then simulated by employing the orientation-averaged Debye equation (the incoherent scattering was discarded since it does not depend on the molecular structure). Supplementary Figure 7 shows the simulated $\Delta S_{XDS}(Q)$ obtained from subtracting the two patterns.

From these considerations, the solute term was expressed as $\Delta S_{solute}(Q,t)=\gamma(t)\cdot\Delta S_{solute}(Q)$, where $\gamma(t)$ is the time-dependent excitation fraction of $[^{2}Ru^{III}={}^{4}Co^{II}(HS)]$.

The solvent term

Assuming the validity of a classical continuum description, the equilibrated state of the solvent can be expressed as a function of two independent hydrodynamical variables chosen as the temperature (T) and the density (ρ). The $\Delta S_{solvent}$ that originates from the bulk-solvent response can be described in terms of their elementary variations $\Delta T(t)$ and $\Delta\rho(t)$. Numerous investigations at synchrotron sources have demonstrated that a first order treatment is adequate to model the response on the hundreds of picoseconds to hundreds of milliseconds time scales. Within this framework the solvent term is quantified through the following linear combination:

$$\Delta S_{solvent}(Q,t) = \Delta T(t)\cdot\left.\frac{\partial S(Q,T)}{\partial T}\right|_{\rho} + \Delta\rho(t)\cdot\left.\frac{\partial S(Q,\rho)}{\partial \rho}\right|_{T} \quad \text{(Supplementary Equation 1)}$$

where $\left.\frac{\partial S(Q,T)}{\partial T}\right|_{\rho}$ and $\left.\frac{\partial S(Q,\rho)}{\partial \rho}\right|_{T}$ are the difference scattering signals arising from a change in T at constant ρ and from a change in ρ at constant T, respectively. These studies have also shown that $\left.\frac{\partial S(Q,T)}{\partial T}\right|_{\rho}$ and $\left.\frac{\partial S(Q,\rho)}{\partial \rho}\right|_{T}$ can be measured independently, making it possible to extract

$\Delta T(t)$ and $\Delta\rho(t)$ directly from the transient $\Delta S_{solvent}$ signals. Simple arguments allow determining the time scales on which the various contributions play a role. Considering first $\Delta\rho(t)$, it has been shown[24] that for times t such that $t < d/v_s$, where d is the FWHM of the laser spot and v_s is the speed of sound in the liquid, no thermal expansion of the solvent has yet taken place. With the present experimental conditions (d =500 µm FWHM and v_s=1280 m/s for MeCN), thermal expansion is expected to happen on the 400 ns time scale, which is beyond the temporal window probed, so that $\Delta S_{solvent}$ reduces to the contribution from impulsive solvent heating :

$\Delta S_{solvent}(Q,t) = \Delta T(t) \cdot \left.\frac{\partial S(Q)}{\partial T}\right|_{\rho}$. The reference difference scattering signal, $\left.\frac{\partial S(Q)}{\partial T}\right|_{\rho}$ (called the temperature solvent differential (TSD)) was acquired independently during a dedicated study at ID09b, ESRF[28]. The data were measured using multilayer optics characterized by a 2.5% bandwidth (bw). Since this is broader than the intrinsic 0.3% bw of the XFEL beam, the influence of the X-ray source spectrum had to be investigated. As a first step, a TSD that would be obtained from a monochromatic beam was constructed from the measurement in ref 28 shown in Supplementary Figure 8A (black trace) after standard filtering (red trace), as described in ref 18. Supplementary Figure 8B shows the resulting curve after Gaussian deconvolution (blue trace). This “monochromatic” TSD (blue) was convoluted with the 2.5% bw of the multilayer (red) and the intrinsic 0.3% bw of an XFEL beam (green). The three curves are indistinguishable, demonstrating that the ID09b reference can be introduced in the analysis of the difference scattering signal from XFEL sources. The convolution of the simulated monochromatic TSD with the full spectrum of the U17 undulator at ID09b (‘pink’ beam) is included for comparison (black trace).

$\Delta S_{XDS}(Q,t)$ from the sample

Summarizing the previous sections, ΔS_{sample} could be expressed as:

$$\Delta S_{sample}(Q,t) = \gamma(t) \cdot \Delta S_{solute}(Q) + \Delta T(t) \cdot \left.\frac{\partial S(Q)}{\partial T}\right|_{\rho} \quad \text{(Supplementary Equation 2)}$$

where $\gamma(t)$ is the fraction of charge-separated molecules, $\Delta S_{solute}(Q)$ is the difference between the signal simulated for the Ru^{II}=$^{1}Co^{III}$(LS) and Ru^{III}=$^{4}Co^{II}$(HS) DFT structures, $\Delta T(t)$ is the change in temperature and $\left.\frac{\partial S(Q)}{\partial T}\right|_{\rho}$ is the change in scattering signal caused by an increase of MeCN temperature at constant density.

Supplementary Note 8: XDS Data Analysis - Fit-based analysis of $\Delta S_{XDS}(Q,t)$

Following the methodology presented by Haldrup[29], each individual difference scattering signal $\Delta S_{XDS}(Q,t)$ was expressed as a sum of a background contribution ($\Delta S_{back}(Q,t)$ = $\sum_{i=1-5} \alpha_i(t) \Delta S^{i}{}_{SVD}(Q)$) and a term $\Delta S_{sample}(Q,t)$ from sample structural changes, both introduced above. For each time delay, the residual function:

$$\Delta S_{res} = \frac{\left(\Delta S_{.}(Q,t) - \Delta S_{sample}(Q,t) - \sum_{i=1-5} \alpha_i(t) \Delta S^{i}{}_{SVD}(Q) \right)^2}{\sigma^2_{XDS}(Q,t)/(N-p-1)} \quad \text{(Supplementary Equation 3)}$$

was minimized. The denominator $\sigma^2_{XDS}(Q,t)/(N-p-1)$ incorporates the point-to-point difference signal noise $\sigma_{XDS}(Q,t)$[25], as well as the number of data points N, and the number of free parameters p in the fit. As such, ΔS_{res} can be directly interpreted as a χ^2 estimator. This allowed extracting the absolute magnitude of every SVD scaling parameters $\alpha_i(t)$, for each time point. While the average value for two of the $\alpha_i(t)$ parameters changed gradually over the duration of the scan, no systematic evolution was observed around or after t = 0. Explicit

inclusion of a density term $\Delta S_{solvent}$ returned $\Delta\rho(t) \approx 0$ for all time delays recorded. Any contribution of the solute-solvent cross-term to $\Delta S_{XDS}(Q,t)$ was below the detection threshold.

Supplementary Note 9: XDS Data Analysis - The heat response of the solvent on ultra-short timescales

The solvent term is derived within a classical hydrodynamic framework that assumes a homogeneous temperature distribution[24]. Following photoabsorption, homogeneity is reached on a time scale that depends on the distance between excited centers. It ranges from ~ 15 ps to ~350 ps for excited state concentrations of 40 mM and 0.3 mM respectively[24,28]. Given the 6 mM * 0.65 = 4 mM concentration of absorbing centers that act as point sources of heat, homogeneity should be restored within ~ 65 ps via thermal diffusion for the experiment presented in this work. On one hand, this shows that the signature of the homogenized temperature distribution has been recorded for the later time points of the covered temporal window. On the other hand, for the earlier times, the influence of these hot spots on $\Delta S_{solvent}(Q,t)$ has to be assessed, and this requires the XDS analysis to enter previously uncharted territory.. The simplified model described below establishes that sizing the signature of inhomogeneous thermalization in $\Delta S_{solvent}(Q,t)$ would necessitate higher temporal resolution than the one that could be achieved in these very first experiments. The argument derives from inspection of simulated difference scattering signals from a series of Molecular Dynamics (MD) calculations that exhibit the same changes in hydrodynamic parameters as those arising from impulsive heating. The calculations were performed in Desmond[30],[31] using the OPLS-aa force field for acetonitrile. These calculations allow extracting the radial pair distribution functions (RPDF) for acetonitrile shown in Supplementary Figure 9. During the calculation of the difference scattering signal from the RPDF generated by the MD

simulations[32], a cut-off value is defined. Beyond this distance the RPDF is damped towards to unity and ceases to contribute to the signal. It was found that the difference scattering arising from the calculated scattering curves reproduced the data measured in ref 28, and were insensitive to the choice of threshold as long as it was larger than 10 Å. This shows that for MeCN the solvent scattering signal arise from the interatomic distances shorter than a characteristic length-scale of about 10 Å, corresponding to the first two solvation shells. This implies that this distance should be used when estimating the criterion for temperature homogeneity as measured by XDS. From Landau and Lifshitz[33] the time scale on which a volume reaches local homogeneity after an impulse temperature change is given by $\tau = l^2/\chi$ where l is the characteristic distance and χ is the thermometric conductivity of the solvent. A distance l = 10 Å leads a homogenization time of 3.1 ps for MeCN. In conclusion, the perturbations to the signal caused by an inhomogeneous temperature distribution are negligible on all but the very shortest times.

Supplementary Note 10: XDS Data Analysis - The linearity of the solvent temperature response

As described in the previous sections the solvent term contribution to the difference scattering signal on the 3 ps – 60 ps timescale is given by an inhomogeneously distributed temperature-increase of the sample. In the previous section it was shown that the temperature-increase is homogeneously distributed on the ~10 Å characteristic length-scale of the acetonitrile RDF. This ‘local’ homogeneity of the temperature-increase ensures that its contribution to the difference scattering can be construed as arising from volume elements having undergone

homogeneous temperature-increase. Thus, the contribution of the temperature-increase to the difference scattering signal:

$$\Delta S(Q,t)_{solvent} = \Delta T(t) \cdot \left.\frac{\partial S(Q)}{\partial T}\right|_{\rho} \quad \text{(Supplementary Equation 4)}$$

can be expressed as:

$$\Delta S(Q,t)_{solvent} = \int_V \Delta T_v(t) \cdot \left.\frac{\partial S(Q)}{\partial T}\right|_{\rho} dv \quad \text{(Supplementary Equation 5)}$$

With $\Delta T_v(t)$ being the temperature change in a given volume element as a function of time and V being the volume of the X-ray/sample interaction volume. As the solvent temperature differential $\left(\left.\frac{\partial S(Q)}{\partial T}\right|_{\rho}\right)$ is a constant reference signal, the integral needs only to be evaluated over $\Delta T_v(t)$:

$$\Delta S(Q,t)_{solvent} = \left.\frac{\partial S(Q)}{\partial T}\right|_{\rho} \cdot \int_V \Delta T_v(t)\, dv \quad \text{(Supplementary Equation 6)}$$

Solving the integral returns an 'average' temperature increase of the solvent ($\Delta T(t)$) even at the 3 ps - 60 ps timescale where a macroscopic homogeneous temperature increase is not yet defined.

Thus on short timescales the 'average' temperature increase returned by the XDS measurement can be construed as a measure for the total increase in solvent temperature had the increase in intermolecular vibrational energy been homogeneously distributed throughout the X-ray/sample interaction volume.

Supplementary Note 11: XDS Data Analysis – The sensitivity of the XDS measurement on the structural dynamics

The XDS analysis relies on fixing the structures of [$^1Ru^{II}$=$^1Co^{III}$] and [$^2Ru^{III}$=$^4Co^{II}$] as obtained from DFT optimizations. Based on these DFT structures, the XDS analysis focuses

on extracting the excited state fraction and the rate of solvent heating. The most significant structural difference between [$^1Ru^{II}$=$^1Co^{III}$] and [$^2Ru^{III}$=$^4Co^{II}$] is the 0.2 Å bond length elongation of the Co-N bond. In order to assess the sensitivity of the XDS measurement to the structure of [$^2Ru^{III}$=$^4Co^{II}$], the difference scattering signal $\Delta S_{XDS}(Q)$ has been simulated for a set of [$^2Ru^{III}$=$^4Co^{II}$] structures where the Co-N bond length elongation relative to the [$^1Ru^{II}$=$^1Co^{III}$] structure – ΔR(Co-N) – was varied between 0.15Å and 0.25 Å. The results are displayed in Supplementary Figure 10. The left panel presents the simulated $\Delta S_{XDS}(Q)$ on an absolute scale, showing that the amplitude of the signal is very dependent on ΔR(Co-N), increasing with larger structural changes: thered curve is simulated for ΔR(Co-N) = 0.15Å, the purple curve simulated for ΔR(Co-N) = 0.25Å). The right panel presents the different simulated $\Delta S_{XDS}(Q)$ curves normalized to the signal amplitude, showing that the *shape* of the simulated $\Delta S_{XDS}(Q)$ are very similar for different ΔR(Co-N).

The observation that the variation of the simulated $\Delta S_{XDS}(Q)$ curves with ΔR(Co-N) is almost exclusively one of signal *amplitude* entails that it will be strongly correlated with the excitation fraction (γ_{XDS}) in the analysis of the XDS data. Due to this correlation significant differences in the excited structure of [$^2Ru^{III}$=$^4Co^{II}$] at early times will be manifested in the $\Delta S_{XDS}(Q,t)$ fits as deviations in γ_{XDS}. Since γ_{XDS} determined for the 3 ps time-delay (0.63 ± 0.2) is well within the uncertainty of the final γ_{XDS} (0.67 ± 0.04), no significant deviation in the excited state structure could be detected for these measurements, even at the earliest time-scales.

Supplementary Note 12: Preliminary QM/MM (solute/solvent) equilibrium MD simulations

The simulations were made using our QM/MM MD implementation in the Grid-based Projector Augmented Wave code (GPAW).[34] The system was comprised of the ruthenium-cobalt dyad, modeled using PBE with a real space grid spacing of 0.18 Å and a dzp LCAO basis, and 436 MM acetonitrile molecules in a 32x32x44 Å box, which was thermalized using a Langevin thermostat on the MM solvent only. The [$^{1}Ru^{II}$=$^{1}Co^{III}$(LS)] ground state was sampled for 18 ps and the [$^{2}Ru^{III}$=$^{4}Co^{II}$(HS)] charge separated state, where the Co^{II} center is in the HS state was allowed to re-equillibrate for 8 ps before sampling for 10 ps, using 1 fs time steps. Supplementary Figure 11A show the thermal distributions for the individual Co-N bond lengths of [$^{1}Ru^{II}$=$^{1}Co^{III}$(LS)] and [$^{2}Ru^{III}$=$^{4}Co^{II}$(HS)] , while Supplementary Figure 11B displays the corresponding thermal distributions of average Co-N.

These simulations confirm the large structural rearrangements that take place around the Co center (ΔR ~ 0.15 Å) following photoinduced ET.

Supplementary References:

[1] Gawelda, W. *et al*, Ultrafast Nonadiabatic Dynamics of $[Fe^{II}(bpy)_3]^{2+}$ in Solution. *J. Am. Chem. Soc.* **129**, 8199–8206 (2007).

[2] Vankó, G. *et al.* Probing the 3d Spin Momentum with X-ray Emission Spectroscopy: The Case of Molecular-Spin Transitions, *J. Phys. Chem. B* **110**, 11647–11653 (2006).

[3] Vankó, G. *et al.*, Picosecond Time-Resolved X-Ray Emission Spectroscopy: Ultrafast Spin-State Determination in an Iron Complex. *Angew. Chem. Int. Ed.* **49** , 5910–5912 (2010).

[4] Rueff, J. P. *et al.*, Magnetism of Invar alloys under pressure examined by inelastic x-ray scattering, *Phys. Rev. B* **63**, 132409 (2001).

[5] Vankó, G., Renz, F., Molnár, G., Neisius, T., Kárpáti, S., Hard-X-ray-Induced Excited-Spin-State Trapping, *Angew. Chem. Int. Ed.* **46**, 5306-5309 (2007).

[6] Sikora, M., Knizek, K., Kapusta, C., Glatzel, P., Evolution of charge and spin state of transition metals in the $LaMn_{1-x}Co_xO_3$ perovskite series, *J. Appl. Phys.* **103**, 07C907 (2008).

[7] Yamaoka, H. *et al.*, Bulk electronic properties of $FeSi_{1-x}Ge_x$ investigated by high-resolution x-ray spectroscopies, *Phys. Rev. B* **77**, 115201(2008).

[8] Herrero-Martín, J. *et al.*, Hard x-ray probe to study doping-dependent electron redistribution and strong covalency in $La_{1-x}Sr_{1+x}MnO_4$, *Phys. Rev. B* **82**, 075112 (2010).

[9] Mao, Z. *et al*, Electronic spin and valence states of Fe in $CaIrO_3$-type silicate post-perovskite in the Earth's lowermost mantle, *Geophys. Res. Lett.* **37**, L22304 (2010).

[10] Rovezzi, M., Glatzel, P., Hard x-ray emission spectroscopy: a powerful tool for the characterization of magnetic semiconductors, *Semicond. Sci. Tech.*, **29**, 023002 (2014).

[11] Németh, Z. *et al.*, Microscopic origin of the magnetoelectronic phase separation in Sr-doped $LaCoO_3$, *Phys. Rev. B*, **88**, 035125 (2013).

[12] Vankó, G.; Rueff, J.-P.; Mattila, A.; Németh, Z. & Shukla, A., Temperature- and pressure-induced spin-state transitions in $LaCoO_3$, *Phys. Rev. B*, 73, 024424 (2006).

[13] Neese, F., The ORCA program system, *WIREs Comput. Mol. Sci*. **2**, 73–78 (2012).

[14] Neese, F., ORCA, version 2.8; (Max- lanck-Institut für Bioanorganische Chemie: Mülheim an der Ruhr, Germany, 2004)

[15] Reiher, M., Salomon, O., Hess, B. A., Reparametrization of hybrid functionals based on energy differences of states of different multiplicity, *Theor. Chem. Acc*. **107**, 48–55 (2001).

[16] Paulsen, H; Trautwein, A. X., Density Functional Theory Calculations for Spin Crossover Complexes, *Top. Curr. Chem*. **235**, 197–219 (2004).

[17] Hauser, A.et al., Low-temperature lifetimes of metastable high-spin states in spin-crossover and in low-spin iron(II) compounds: The rule and exceptions to the rule, *Coord. Chem. Rev.* **250**, 1642–1652 (2006**).**

[18] Vargas, A., Zerara, M., Krausz, E., Hauser, A., Daku, L. M. L., Density-Functional Theory Investigation of the Geometric, Energetic, and Optical Properties of the Cobalt(II)tris(2,2‘-bipyridine) Complex in the High-Spin and the Jahn−Teller Active Low-Spin States, *J. Chem. Theory Comput*., **2**, 1342–1359 (2006**)**.

[19] Shiota, Y., Sato, D., Juhasz, G., Yoshizawa, K. J., Theoretical study of thermal spin transition between the singlet state and the quintet state in the $[Fe(2\text{-picolylamine})_3]^{2+}$ spin crossover system. *Phys. Chem. A*, **114**, 5862 –5869 (2010**)**.

[20] Papai, M., Vanko, G., de Graaf, C., Rozgonyi, T., Theoretical Investigation of the Electronic Structure of Fe(II) Complexes at Spin-State Transitions, *J. Chem. Theory Comput.*, ***9***, 509–519 (2013**)**.

[21] Klamt, A.,; Schüürmann, G., COSMO: a new approach to dielectric screening in solvents with explicit expressions for the screening energy and its gradient, *J. Chem. Soc., Perkin Trans.* **2**, 799–805 (1993).

[22] Cromer, D. T., Mann, J. B., X-ray scattering factors computed from numerical Hartree-Fock wave functions, *Acta Cryst. A* **24**, 321–324 (1968).

[23] Hajdu, F., Revised parameters of the analytic fits for coherent and incoherent scattered X-ray intensities of the first 36 atoms, *Acta Cryst. A* **28**, 250–252. (1972).

[24] Cammarata, M. *et al*., Impulsive solvent heating probed by picosecond x-ray diffraction., *J. Chem. Phys*. **124**, 124504 (2006).

[25] Haldrup, K., Christensen, M. & Meedom Nielsen, M., Analysis of time-resolved X-ray scattering data from solution-state systems, *Acta Cryst. A* **66**, 261−269 (2010).

[26] Becke, A. D., Density-functional exchange-energy approximation with correct asymptotic behavior., *Phys. Rev. A* **38**, 3098−3100 (1988).

[27] Perdew, J. P., Density-functional approximation for the correlation energy of the inhomogeneous electron gas., *Phys. Rev. B* **33**, 8822−8824 (1986).

[28] Kjær, K. S. *et al.*, Introducing a standard method for experimental determination of the solvent response in laser pump, X-ray probe time-resolved wide-angle X-ray scattering experiments on systems in solution, *Phys. Chem. Chem. Phys.* **15**, 15003−15016 (2013).

[29] Haldrup, K., Singular value decomposition as a tool for background corrections in time-resolved XFEL scattering data, *Philos. Trans. R. Soc. London, Ser. B*, **369**, 20130336 (2014).

[30] *Schrödinger Release 2013-1: Desmond Molecular Dynamics System, version 3.4,* D. E. Shaw Research, New York, NY, (2013).

[31] *Maestro-Desmond Interoperability Tools, version 3.4,* Schrödinger, New York, NY, (2013).

[32] Ihee, H. *et. al.,* Ultrafast X-ray scattering: structural dynamics from diatomic to protein molecules, *Int. Rev. Phys. Chem.* **29**, 453−520 (2010).

[33] Landau, L. D., Lifshitz, E. M., *Fluid Mechanics* (Pergamon Press, Oxford, ed. 2, 1987).

[34] Dohn, A. O. *et al.*, Direct Dynamics Studies of a Binuclear Metal Complex in Solution: The Interplay Between Vibrational Relaxation, Coherence, and Solvent Effects, *J. Phys. Chem. Lett.* **5**, 2414−2418 (2014).
